# A model-based approach to study ant energetics from trajectory data

**DOI:** 10.1093/pnasnexus/pgag174

**Published:** 2026-05-28

**Authors:** Basit Yaqoob, Michael Napoli, Nicola Pugno, Maurizio Porfiri

**Affiliations:** Center for Urban Science and Progress, Tandon School of Engineering, New York University, Brooklyn, NY 11201, USA; Department of Mechanical and Aerospace Engineering, Tandon School of Engineering, New York University, Brooklyn, NY 11201, USA; Department of Mechanical Engineering, National University of Technology, Islamabad 44000, Pakistan; Center for Urban Science and Progress, Tandon School of Engineering, New York University, Brooklyn, NY 11201, USA; Department of Mechanical and Aerospace Engineering, Tandon School of Engineering, New York University, Brooklyn, NY 11201, USA; Mechano-X Labs, Department of Civil, Environmental and Mechanical Engineering, University of Trento, Trento 38123, Italy; School of Engineering and Materials Science, Queen Mary University of London, London E1 4NS, United Kingdom; Center for Urban Science and Progress, Tandon School of Engineering, New York University, Brooklyn, NY 11201, USA; Department of Mechanical and Aerospace Engineering, Tandon School of Engineering, New York University, Brooklyn, NY 11201, USA; Department of Civil, Urban, and Environmental Engineering, Tandon School of Engineering, New York University, Brooklyn, NY 11201, USA; Department of Biomedical Engineering, Tandon School of Engineering, New York University, Brooklyn, NY 11201, USA

**Keywords:** cost of transport, energy consumption, insect biomechanics, locomotion, video data

## Abstract

Whether an insect belongs to a solitary or a social species, its survival depends on how it consumes available energy resources. The characterization of energy consumption has largely relied on flow respirometry, a technique that is difficult to implement on individuals in isolation and is ill-conceived to disaggregate energy costs among individuals in a colony. Toward addressing these issues, we introduce an integrative framework that combines the growing area of video tracking with rigorous biomechanical modeling to infer locomotion energetics in ants. Our approach rests upon a novel physics-based model for hexapedal locomotion in the sagittal plane, which predicts forward motion and vertical oscillations in terms of few model parameters that can be retrieved from the literature or mathematically constrained. We show that individual ants favor adaptability over energy efficiency by incorporating higher step frequencies and rely less on energy recovery. A mechanistic approach to input energy estimation, combined with biomechanical analysis, provides a valuable toolkit for biologists to investigate ant energetics and perform informative inter-species comparison, from trajectory data.

Significance Statement Video-tracking is transforming the way behavioral observations are performed on insects, enabling the precise quantification of complex behavioral repertoires from the laboratory to the field. Could these datasets help shed light on energy consumption of insects in groups or isolation? In search for an answer to this question, we put forward a model of hexapedal locomotion that links locomotion kinematics acquired from video data with energy consumption. Grounded in first physical principles, our approach bestows generalizable and interpretable insights for researchers to draw reliable inferences on ant energetics.

## Introduction

Energy is a fundamental resource for insects, shaping their growth, reproduction, and activity patterns (1). Understanding energy usage in insects is key to shed light on life-history optimization at the individual level (2) and to investigate macroecological patterns, such as distribution and abundance in the environment (1). In eusocial insects, energy regulation largely takes place at the colony level, where selective pressures favor superorganism-like coordination (3). Energy availability during the founding stage filters colony success under environmental constraints (4). As the colony matures, energy allocation shifts from growth to maintenance, where larger colonies exhibit lower mass-specific metabolic rates, slower growth, and reduced reproduction that favor longevity (3). Polymorphism adds another layer of energetic regulation in social insects, where the activity of smaller versus larger workers is regulated to optimize collective energy expenditure (5).

Flow respirometry is the cornerstone for measuring energy consumption in insects (1). Flow respirometry entails monitoring the flow of air through a chamber containing one or multiple insects and quantifying changes in oxygen concentration that serve as a measure of energy consumption. This technique has been successfully applied to estimate the energy costs of rafting, walking, foraging, flying, thermo-regulation, and burrowing in insects (1, 6–11); yet, its use for small insects is challenged by technical limitations, like leakage, water vapor interference, and flow rate variations (12). For small insects, standard practice recommends push-mode carbon dioxide respirometry in which controlled and measured air is pushed into the chamber before it interacts with the insect (12). An alternative approach is constituted by the Clark electrode system, in which one measures the electric current produced as oxygen diffuses across a permeable membrane and is reduced at a platinum cathode (13). The Clark method has been successfully used for measuring oxygen consumption in individual insects with high temporal resolution and robustness against discontinuous gas exchange-cycle (DGC), although chamber sealing requirements and polymerization wait times significantly constrain experimental throughput (14). Also, the accurate interpretation of respirometry data is challenged by the complexity and variability of insect respiratory patterns, which include diverse spiracular phases (closed, flutter, and open) and rhythmic tracheal compression, coupled with significantly different timescales of tracheal collapse and abdominal compressions (12, 15).

Overcoming the technical limitations of flow respirometry will certainly improve the accuracy and flow resolution for the measurement of single insects, yet, it does not provide a pathway for understanding the role of social interactions on energy regulation at the colony level for eusocial insects. In fact, flow respirometry only outputs a single measurement for the change in oxygen in the chamber, without offering any means to disaggregate individual energy costs. Are larger individuals consuming more energy than smaller ones? How much does caring of the brood cost energetically to the colony? How much energy is spent on the maintenance tasks?

Automated video tracking has emerged as a promising tool for the study of insect behavior, allowing for pose estimation and trajectory estimation (16). Deep learning models, such as YOLO and ResNet, have enhanced detection accuracy, while tracking algorithms, like the particle-Kalman Filter and graph-based identity resolution, address the challenges of occlusion and interaction. Background subtraction, feature extraction, and 3D pose estimation further enhance the analytical depth, making video tracking a scalable and noninvasive tool for activity tracking. To track individual insects in complex environments, color- and motion-based machine vision are combined, and nighttime video analysis reveals individual’s variation in spatial exploration and foraging rhythms (17, 18). Convolutional neural networks, including YOLOv8, have been trained to detect ant colonies and leaf transport in leaf-cutter ants, informing agroecological management strategies (19, 20). Advances in motion tracking have informed the inference of energy expenditure in fish (21–23) and humans (24–28), yet comparable approaches remain largely unexplored in insects.

Here, we propose a model-based approach to infer energy consumption of single insects from video tracking of their motion. We focus on ants and study energy consumption in terms of their input energy to walk one step, the input energy per unit step (cost of transport [CoT]), and the energy efficiency associated with forward motion. The contribution of our study is 2-fold. First, we develop a physics-based model of hexapedal locomotion using a sagittal plane representation and demonstrate its ability to anticipate realistic walking trajectories. This approach facilitates the comparison of locomotor characteristics between different ant species, providing insights into how morphological traits and ecological demands shape movement strategies (29). Two commonly employed models for hexapedal locomotion are the lateral leg spring (LLS) and the Full-Goldman template (30–32). The LLS model was used to simulate the ground plane dynamics of cockroaches (30–32), while the Full-Goldman template models the climbing dynamics of cockroaches (33). Although recent efforts have produced detailed multibody models of the ant musculoskeletal system (34–36), to the best of our knowledge, no prior study has applied physics-based models to systematically investigate ant locomotion and energy expenditure. Second, we demonstrate the application of the model for the prediction of the CoT from video-based kinematic data. Motion in the sagittal plane is used to extract simple measurements of the center of mass (CoM), which are integrated in the locomotion model to estimate the input energy, CoT, and energy efficiency. The proposed methodology can be used to link morphological (leg length, body mass, body size, and body height), kinematic (forward speed, step length, and step frequency), and energetic (input energy, CoT, and efficiency) quantities from behavioral observations, enabling a mechanistic understanding of insect biomechanics. Such insights may facilitate comparative analyses across species and potentially inform the design of energy-efficient robotic systems (37, 38).

## Results

### Mathematical model

**Figure 1:**
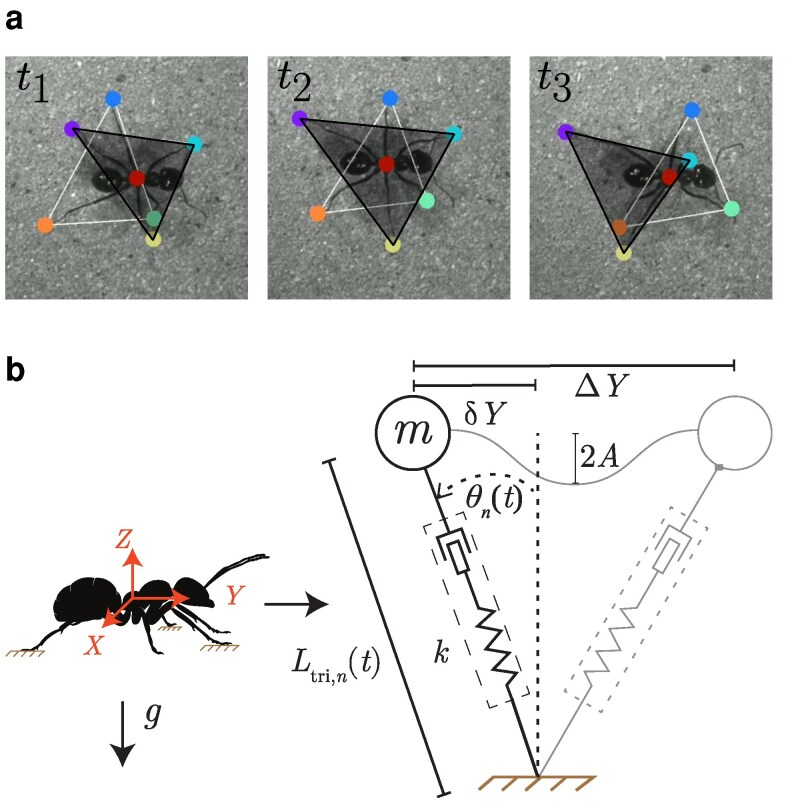
Example of a tripod gait and the schematic of the locomotion model. Snapshots (a) from a video recording by Pfeffer et al. (39) show the tripod gait for a *C. fortis* ant at three points in time: ($t_{1}=0$) the focal tripod (black, shaded triangle) is fully extended forward and makes contact with the ground; ($t_{2}=70\;\text{ms}$) the nofocal tripod (white triangle) and the CoM (red dot) swing forward along the horizontal axis; ($t_{3}=135\;\text{ms}$) the focal tripod is fully extended and the stance transitions as the nonfocal tripod makes contact with the ground. We connect the CoM (b) of the ant (denoted by *m*) to the centroid of the current tripod configuration (denoted by the foot position). The link is extensible with actuation function $L_{\mathrm{tri},n}$, and an opposing force represented by the spring stiffness *k* that mimics the resistance to deformation created by the muscles. The deflection of the CoM from the vertical position is indicated by the angle $\theta_{n}$. δY is the fixed distance between the CoM and the foot position at the start of the cycle. In a step, the CoM covers distance ΔY distance in the forward direction and the amplitude of the vertical oscillations amounts to *A*.

We study ant locomotion in the sagittal plane. We assume that ants employ a tripod gait to propel the body forward, without involving a double-stance or aerial phase (Fig. 1a) (29, 33, 40). The tripod gait consists of two alternating sets of three legs (tripods) that support the CoM during locomotion to maintain balance while walking. When one tripod completes its swing phase, the other completes its stance phase and the two exchange roles. Although ants exhibit brief double-stance phases at low speeds and aerial phases at higher speeds, empirical evidence suggests that grounded running—where at least one leg remains in contact with the substrate—is dominant across their full speed range (41–44). We hypothesize a 50 duty factor in each leg (the duty factor represents the percentage of time that a given tripod is spent in contact with the ground) and we neglect double stance and aerial phases, so that each leg contributes equally to the dynamics of locomotion. Finally, we neglect the effect of surface adhesion created by contact between the body of the ant and the ground, limiting our analysis to ants with elevated postures such as in the *Cataglyphis* genus (45). The tripod assumption parallels hypotheses used in simplified models of bipedal locomotion across a wide range of animal morphologies (30, 46–49).

Given the symmetry of alternating tripod cycles, we focus on a single tripod in our schematic representation (Fig. 1b). The ant’s body is modeled as a point mass, *m*, with positional coordinates *Y* and *Z* along the horizontal and vertical axes, respectively. Each tripod is assumed to be massless, since legs contribute <10 of the total body mass, and the CoM is acted on by the acceleration of gravity *g* (42). To emulate energy changes associated with muscle contraction and relaxation, the tripod is modeled as an extensible spring characterized by a constant spring stiffness *k* and a time-varying equilibrium configuration, which we control through the function of time $L_{\mathrm{eq}}$. Locomotion is thus governed by the force generated from deviations between $L_{\mathrm{eq}}$ and the actual tripod length, $L_{\mathrm{tri}}$, analogous to the spring loaded inverted pendulum model (50). The length of the tripod is constrained by the maximum extent of the leg of the ant, $L_{\max}$, defined as the sum of the basitarsus, tibia, and femur, following the methods of Sommer et al. (45).

In our model (Fig. 1b), the lateral body dynamics are discarded under the assumption of negligible contribution to the total CoM momentum (42). The control function $L_{\mathrm{eq}}$ is constructed so as to emphasize vertical oscillations in the CoM. This modeling choice departs from the literature, where the prototypical model of animal locomotion uses a feed-forward control function on the CoM to create a horizontal, tangential arc in each step (51). For certain ant species, however, the vertical dynamics play a significant role in the dynamics of locomotion. To account for the vertical deviation in the CoM, we adopt similar assumptions to that of Brown et al. (47, 52), where the feed-forward control is replaced with a control function, $L_{\mathrm{eq},n}$, that introduces fixed oscillations.

Our assumptions on the dynamics in the sagittal plane yield the following model equations:


(1a) $$m\ddot{Y}(t)=-k(L_{\mathrm{tri},n}(t)-L_{\mathrm{eq},n}(t))\frac{Y(t)-Y_{f,n}}{L_{\mathrm{tri},n}(t)},$$

(1b) $$m\ddot{Z}(t)=-k(L_{\mathrm{tri},n}(t)-L_{\mathrm{eq},n}(t))\frac{Z(t)}{L_{\mathrm{tri},n}(t)}-mg,$$

where *t* is time, *Y* and *Z* are the forward and vertical positions, respectively, and a superimposed dot denotes a derivative in time. The model is parameterized by the mass *m*, spring stiffness *k*, and gravity *g*. Given that we focus on walking over level terrain, the vertical position of the foot is zero. Subscript *n* labels the step number, and $t_{n}$ is the time at which step *n* begins. The length of the tripod during locomotion, $L_{\mathrm{tri},n}$, is given by the following expression (Fig. 1b):


(2) $$L_{\mathrm{tri},n}(t)=\sqrt{\left(Y(t)-Y_{f,n}{)}^{2}+Z^{2}(t\right)},$$

where $Y_{f,n}$ is the forward position of the foot during step *n*.

The model is actuated through control function, $L_{\mathrm{eq},n}$. Following the methods of Brown et al. (47)—inspired by experimental sinusoidal force trends (33, 42, 49)—we utilize the following function:


(3) $$L_{\mathrm{eq},n}(t)=L_{0}-L_{d}\cos\;(\omega (t-t_{n})).$$

Similar to Brown et al. (47), the actuation function is parameterized by the most recent step event. The length of the tripod at rest (and in the absence of gravity) is denoted by $L_{0}$, the radian frequency of the actuation by *ω*, and the amplitude of oscillations by $L_{d}$. In general, control function [3] can take negative values, corresponding to the spring pushing away from the ground plane irrespective of where the ant body is located with respect to the foot.

Next, we define the behavior of the model at each stance transition. The time of the next step is determined by boundary condition


(4) $$t_{n+1}=\min\left\{t>t_{n}\;:\;\dot{Y}(t)=\dot{Y}(t_{n})\right\},$$

where $t_{n+1}-t_{n}$ is the step period, which at steady state converges to Δt. We rely on the periodicity of the forward velocity to find a suitable trajectory for the sagittal plane motion. At the time of the step, we also define the forward position of the foot with respect to the current position of the CoM as


(5) $$Y_{f,n+1}=Y(t_{n+1})+\delta Y,$$

where δY is the step size, a parameter that defines the position of the pivot point in relation to the forward position of the CoM. The step size is generally different from the step length which is the forward distance covered in a step period and is denoted as ΔY at the steady state (Fig. 1b).

By nondimensionalizing [1], we identify a small set of fundamental parameters. We choose the inverse of the actuation radian frequency as the characteristic time scale and define nondimensional time, *τ*, through $t=\frac{\tau }{\omega }$. We use $L_{0}$ as the characteristic length scale, such that $L_{\left(\cdot \right)}=L_{0}\ell_{\left(\cdot \right)}$, $Z=L_{0}z$, and $Y_{\left(\cdot \right)}=L_{0}y_{\left(\cdot \right)}$. This choice allows us to eliminate $L_{0}$ from the nondimensional formulation, thereby simplifying the analysis. Finally, we use *m* as the characteristic mass. For simplicity, we omit index *n* from the model, as we only consider trajectories at steady state which are periodic. Hence, we write


(6a) $$\ddot{y}(\tau )=-\varphi^{2}(\ell_{\mathrm{tri}}(\tau )-\ell_{\mathrm{eq}}(\tau ))\frac{y(\tau )-y_{f}}{\ell_{\mathrm{tri}}(\tau )},$$

(6b) $$\ddot{z}(\tau )=-\varphi^{2}(\ell_{\mathrm{tri}}(\tau )-\ell_{\mathrm{eq}}(\tau ))\frac{z(\tau )}{\ell_{\mathrm{tri}}(\tau )}-\gamma ,$$

where $\ell_{\mathrm{tri}}(\tau )=\sqrt{(y(\tau )-y_{f}{)}^{2}+(z(\tau ){)}^{2}}$, and $\varphi =\frac{\omega_{0}}{\omega }$ and $\gamma =\frac{g}{L_{0}\omega^{2}}$ are dimensionless constants, and $\omega_{0}=\sqrt{\frac{k}{m}}$ is the natural frequency of the mass-spring system. The frequency ratio, φ, summarizes the relationship between the natural frequency of the tripod spring and the frequency of the effective stepping force. When φ=1, the two are equal and the dynamics of the pendulum are prone to resonate: predicting large, difficult-to-control oscillations in the CoM over the walking period (53). When φ<1, the actuation frequency dominates the CoM dynamics and for certain values of *γ* we can expect the emergence of a stable walking solution with a gait period proportional to the actuation frequency, $\Delta t\approx \frac{2\pi }{\omega }$.

Initial conditions are denoted as $z(0)=z_{0}$, $\dot{z}(0)={\dot{z}}_{0}$, and $\dot{y}(0)={\dot{y}}_{0}$ (see “Numerical simulations” section for details on numerical simulation and Supplementary information I for a sample trajectory). Parameter *γ* is proportional to the inverse of the Froude number, which is the ratio between the gravitational force to the inertial force (54). To nondimensionalize the actuation function, we simply write $\ell_{\mathrm{eq}}(\tau )=1-\ell_{d}\cos\;(\tau )$. Similarly, the nondimensional step size is written as δy, the nondimensional step length as Δy, and the nondimensional step period as Δτ. We highlight that δy describes the position of the pivot at the point of each transition, and Δy the length of space covered over the step period. Using the step period and the actuation frequency, we retrieve the step frequency measured in Hz, as $f=\frac{\omega }{\Delta \tau }$.

We define the feasible region as the area in the plane of the frequency ratio, φ, and inverse Froude number, *γ*, where the body achieves rhythmic walking while satisfying that z(τ)>0 for all *τ*, that is, the body stays above the ground. The feasible region contains a wide range of mechanically plausible postures in which the body adjust oscillations in the CoM based on the parameters φ and *γ*. In biological terms, it reflects how hexapedal insects adapt their posture to accommodate changes in locomotion parameters (see “Determination of a feasible region” section for details on the construction of the feasible region).

A common simplification in the locomotion literature is to neglect CoM oscillations along the vertical axis, that is, $a=\frac{1}{2}(\max(z(\tau ))-\min(z(\tau )))\ll 1$ (in dimensional form, *A*) (30, 31, 51). Following this line of approach, we examine an approximation of model [6] to capture momentum transfer in organisms whose forward position dominates CoM oscillations during locomotion (see “Simplified model” section for details).

Given a feasible CoM trajectory, parameterized by a (φ,γ)-pair, the motion along the *y*- and *z*-axes can be translated into energetic terms by evaluating both the energy input to the system and the energy transferred into forward propulsion. We specifically estimate: the force exerted through the leg, $f_{\mathrm{leg}}$, energy input, $e_{\mathrm{in}}$, CoT, percent efficiency of locomotion, and percent congruity (see “Energy metrics” section for details).

### Model predictions about locomotion features

**Figure 2:**
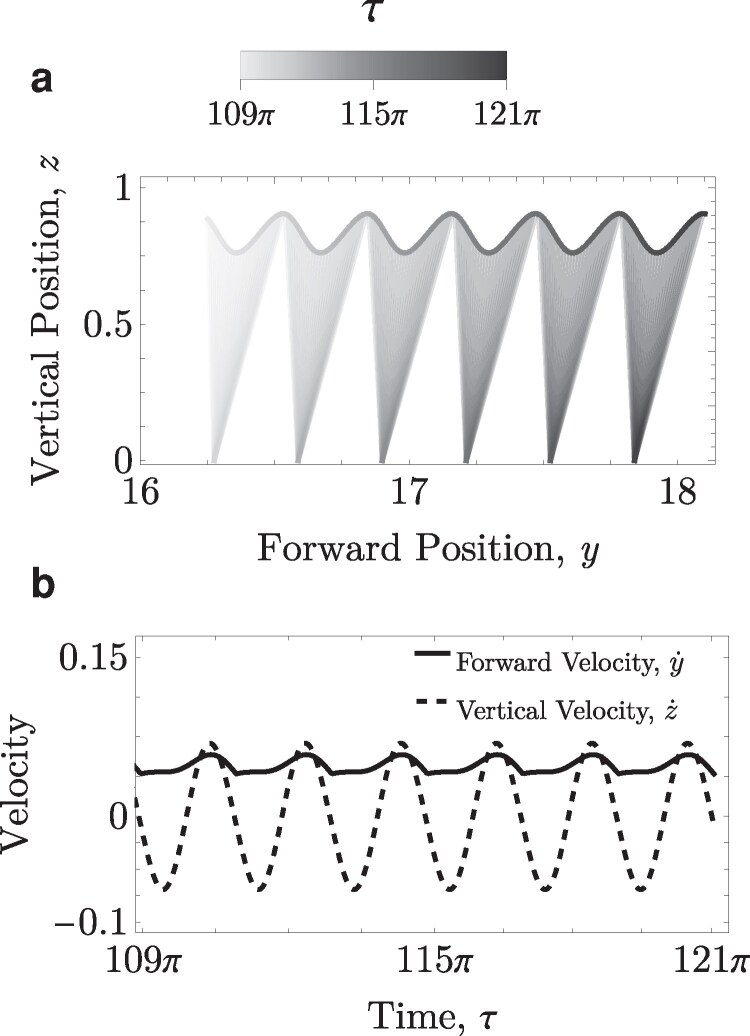
Example walking trajectory in the sagittal plane. In a) we demonstrate six steps of the sagittal plane model for parameter conditions $\ell_{d}=1.5$, φ=0.204, γ=0.0041, δy=0.05, $z_{0}=1$, ${\dot{y}}_{0}=0.04$, and ${\dot{z}}_{0}=0$. The vertical and horizontal axes represent the nondimensional height, *z*, and forward position, *y*, of the CoM, respectively, while the shading of the CoM and superimposed tripod positions denote the passage of nondimensional time, *τ*. In b), we show the vertical, $\dot{z}$, and horizontal, $\dot{y}$, velocity for the same time window.

Figure 2 illustrates a representative trajectory of the sagittal plane model [6] for a single set of initial and parameter conditions. The motion in the sagittal plane exhibits characteristic periodic vertical oscillations while maintaining a nearly constant forward velocity (Fig. 2a and Supplementary Video 1, whose description is in Supplementary information I). The vertical velocity varies significantly over the gait cycle, in contrast to the relatively steady forward speed (Fig. 2b). Given that time is scaled by the inverse of the actuation radian frequency, the period of the walking cycle is ∼2π for all observed trajectories.

The frequency ratio, φ, and inverse Froude number, *γ*, govern the phenotype of the trajectories of locomotion (see Supplementary information I for a detailed analysis). While the forward speed scales directly with both φ and *γ*, these parameters have different effects on body dynamics that are revealing of distinct biomechanical strategies. Increases in φ can be obtained through one of two mechanisms in an ant with fixed mass—either through an increase in the spring stiffness or a decrease in the step frequency. Under the former mechanism, the ant is stiffening the legs, allowing the body to adopt a more erect posture with amplified CoM oscillations. Under the latter mechanism, the ant is increasing the speed at which it oscillates its legs—causing a likewise increase in the amplitude and posture height. Increases in *γ* are observed through a decrease in either the rest length of the tripod (a flatter posture) or the oscillation frequency (a slower walk). In either case, the mean height of the CoM during the trajectory decreases without significantly influencing its amplitude, resembling a transition to a crouch-like posture.

**Figure 3:**
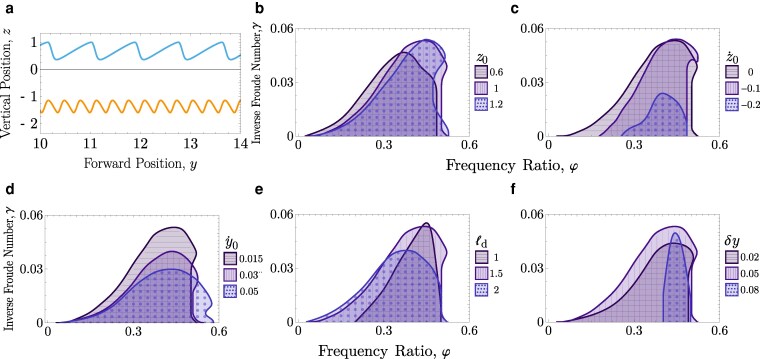
Extent of the feasibility region for changing initial conditions and model parameters. Feasible walking regions are constructed by simulating [6] to steady state (a) and checking that the gait satisfies the stability and trajectory conditions described in “Determination of a feasible region” section. Stability alone is not sufficient for describing a feasible walking trajectory as the CoM must also oscillate above the horizontal axis (blue trajectory); otherwise the trajectory is considered infeasible (yellow trajectory). In each feasibility region, simulations are computed with $z_{0}=1$, ${\dot{z}}_{0}=0$, ${\dot{y}}_{0}=0.015$, $\ell_{d}=1.5$, and δy=0.05 unless denoted as the variable of interest—in which case their value is given by the legend on the right of each subfigure. In b), we show that starting the gait with an erect posture (increasing $z_{0}$) enhances the resilience of the locomotion to gravitational effects that is the height of the feasible region increases. Similarly, increasing the initial downward speed (c) decreases the adaptability of the locomotion toward increasing gravitational effects. We find that increases in the initial forward velocity (d, ${\dot{y}}_{0}$) decrease the area of the feasibility region along the axis of *γ*. The area of the feasibility region is inversely proportional to the actuation ratio (e, $\ell_{d}$). The effect of the step size, δy, is shown in f), where we note that increases in the magnitude of δy changes the area coverage in the domain of φ and *γ*.

We begin our analysis of locomotion by computing the feasible region described in “Determination of a feasible region,” section thereby identifying parameter ranges that yield motion consistent with ant locomotion. The feasible walking region is defined as the subset of the parameter space which yields: (i) numerically stable walking trajectories, (ii) a positive and periodic vertical position, (iii) a periodic vertical velocity, and (iv) a positive and periodic forward velocity. Figure 3 demonstrates the feasible walking region and how variations in each of the initial conditions, as well as the model parameters ($\ell_{d}$ and δy), influence its geometry. A larger area of the feasible region indicates that an extensive repertoire of gait patterns can be achieved, thus representing the adaptability of walking by varying muscle actuation. Since the states of [6] are nonlinearly coupled, initial conditions play a role in determining the step dynamics at steady state directly.

We find that insects can increase their adaptability toward gravitational effects by starting the walk with an erect posture. This phenomenon is captured by the increase in the height of the feasible region (Fig. 3a). The other way to increase the adaptability of the body is to start the walk with a null initial downward velocity, as indicated by the highest attainable region area (Fig. 3b). Beginning from a positive vertical velocity, the system does not converge to a stable walking trajectory for any φ and *γ* values. This inability to stabilize the CoM trajectory is likely due to the initial over-extension of the tripod spring, which causes the CoM to accelerate toward the ground axis in phase with the changing equilibrium length; prompting us to only consider negative vertical initial velocity. Increasing ${\dot{y}}_{0}$ raises the stepping frequency without inducing the vertical oscillations required to support the body; large increases in the initial forward velocity ultimately cause the body to fall (Fig. 3c). Thus, decreasing the initial forward velocity increases the feasible range of *γ* and that the system can compensate for larger nondimensional gravitational affects on the CoM. Increasing the actuation amplitude, $\ell_{d}$, decreases the extent of the feasible region along both the of φ- and *γ*-axes (Fig. 3d). We also note that an increase the step size, δy, significantly impacts the shape of the region (Fig. 3e).

By analyzing the influence of initial conditions and actuation parameters on the gait metrics (CoM amplitude, step length, forward speed, input energy, and step frequency), we find that the actuation amplitude, $\ell_{d}$, linearly influences the CoM amplitude, while initial forward velocity linearly affects the step length (see Supplementary information I for details). On the other hand, δy has a secondary effect on the gait metrics (see Supplementary information I for details). Similarly, gait metrics are insensitive to initial height and vertical velocity (see Supplementary information I for details). These sensitivity analysis can be used to find species relevant initial conditions and actuation parameters.

**Figure 4:**
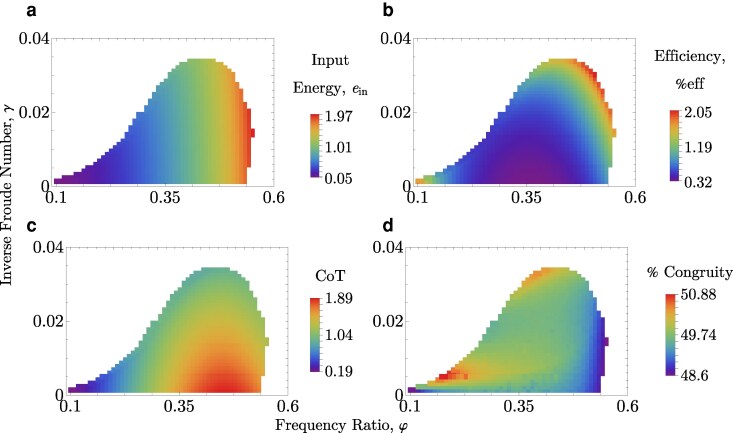
Nondimensional gait metrics plotted over the feasibility region as functions of the parameters φ and *γ*. We hold all other parameters constant at values of $z_{0}=1$, ${\dot{z}}_{0}=0$, ${\dot{y}}_{0}=0.04$, $\ell_{d}=1.5$, and δy=0.05. Gait energy (a) and efficiency correlate strongly with one another and increases along the axis of φ and *γ*. The CoT (c) demonstrates an inverse relationship with efficiency and gait energy. Percent congruity (d) is more or less constant over the entirety of the feasibility region with value of ∼50.

With the feasible region defined, we apply the energy metrics developed in “Energy metrics” section to evaluate the influence of φ and *γ* on the magnitude of these metrics. The system reveals an energy-efficiency trade-off: increasing energy expenditure (Fig. 4a) leads to proportionally higher locomotor efficiency (Fig. 4b) and a reduction in the CoT (Fig. 4c), driven by increased natural frequency associated with leg stiffening. Notably, this aligns with empirical observations suggesting that ants exhibit low energy recovery during locomotion (42, 55). Unlike larger animals that often exhibit high elastic energy storage and return, ants seem to rely less on the pendular or spring-like energy exchange during walking (56, 57). In fact, the minimum CoT (MCoT) is found to occur where the step length is maximized, despite the increase in vertical oscillations (see the gait metrics computed in Supplementary information I). This finding coincides with observations on human walkers, where it has been found that CoM oscillations provide a mechanism of energy exchange which reduces metabolic cost (58, 59). Percent congruity (Fig. 4d) remains relatively constant across the feasible region, indicating that kinetic and potential energies are approximately in phase for half the gait cycle, regardless of φ and *γ*. A percentage congruity of ∼50 suggests that the kinetic and potential energies are capable of exchanging energetics through the pendular mechanism, creating a path for energy recovery during locomotion.

### Inference of energy consumption

**Figure 5:**
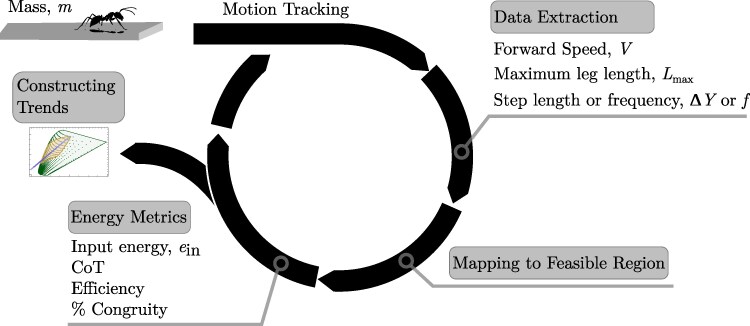
Framework for estimating input energy in a target ant species. The method is broken into three steps, which can be replicated to extract species-level trends. First, the locomotion of an individual ant of mass *m* is measured from the sagittal plane view and the mass is either estimated, or measured explicitly. Second, the mean body height, step frequency and speed are extracted from the videos using modern image processing techniques. Third, the point is mapped onto the feasible region by rescaling the region to the forward speed versus step length and frequency domains.

Having established the ability to predict walking trajectories in a nondimensional, morphology agnostic system, we now aim to establish an experimental framework to connect simple locomotion measurements taken on individuals to input energy. To estimate input energy from video data, one can extract the ant’s forward speed and step length, then use the length of the leg, measured from an ant, to rescale the feasible region and input energy values (Fig. 5). The methodology is deliberately simple, relying solely on the forward speed and step length or frequency of the freely moving ant. Unlike flow respirometry, this approach only requires image processing.

The experimental process can be broken into three steps: (i) the ant is tracked in the sagittal plane for some period of time, (ii) the forward speed, *V*, maximum leg length, $L_{\max}$, and step length, ΔY (or step frequency, *f*, depending on what the experimenter considers to be more reliable) are extracted from the videos, and (iii) the data point is mapped on the feasible region assuming knowledge of other parameters, yielding an estimate of the energy of locomotion from the dynamics of [6]. This process is depicted in Fig. 5. As discussed, the extent of the feasible region has a complex dependency on ${\dot{y}}_{0}$, δy, and $\ell_{d}$, after an extensive parametric analysis, however, we determine that the parameter combination $\left(z_{0},{\dot{z}}_{0},{\dot{y}}_{0},\ell_{d},\delta y)=(0,1,0.015,1.5,0.05\right)$ yield locomotion dynamics relevant to ants (see the sensitivity analysis in Supplementary information I). Note that these parameters correspond to those chosen in Fig. 4, so that the experimenter can extract all energy metrics in their nondimensional form from knowledge of nondimensional parameters *γ* and φ from Fig. 4. Should one have access to direct or indirect measurements of actuation amplitude, step size, and initial forward velocity, they could use them in the model for more accurate predictions of energy expenditure.

**Figure 6:**
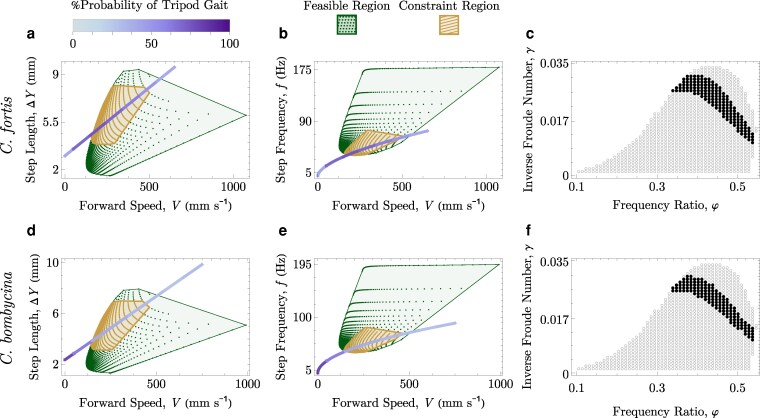
Comparison between experimental trends describing forward speed versus step length (a, d) and step frequency (b, e), with superimposed predictions from the rescaled sagittal plane model (29). At each point in the feasible region, we compute the forward speed, step length, and frequency corresponding to that (φ,γ)-pair. Then, using maximum leg lengths, $L_{\max}$, of 8.25mm and 7mm for *C. fortis* (a–c) and *C. bombycina* (d–f), respectively, we rescale each point in the nondimensional region to the forward speed versus step length (a, d) and frequency (b, e) domain. The proposed model is capable of predicting step length and frequency (a, b) in *C. fortis* ants for forward speeds greater than $∼158\;\text{mm}\;{\text{s}}^{-1}$. To depict the range of φ and *γ* which best represent the ant species, we extract the points lying nearest the experimental trend line and superimpose them onto the feasible region. In *C. bombycina* ants, the overlapping region (d, e) begins at $∼141\;\text{mm}\;{\text{s}}^{-1}$. The overlapping region (f) is in good agreement with that of *C. fortis* ants, satisfying expectations based on the similar morphological characteristics of the two species. The shades in the trend line show the probability of the occurrence of the tripod gait across different speed ranges; these probabilities are computed from the relative frequency of the various gaits, which we measure from the experiments reported by Pfeffer et al. (29).

To demonstrate the use of the proposed method, we use forward speed, step length, and step frequency data on *Cataglyphis fortis* and *Cataglyphis bombycina* ants from Pfeffer et al. (29) with estimates of the mass of the individuals. In the cited work, the authors extracted forward speed, step length, and step frequency data across multiple runs of ants. We infer the leg length from maximum leg lengths of $L_{\max}=8.25\;\text{mm}$ for *C. fortis* and $L_{\max}=7\;\text{mm}$ for *C. bombycina* (45). The feasible region is then superimposed onto the plane of forward speed versus step length (Fig. 6a and d) and step frequency (Fig. 6b and e). Then, we superimpose the speed-step trends extracted by Pfeffer et al. (29) onto the feasible region (Fig. 6c and f). The largest overlap with experimental trends is determined by varying the nondimensional initial forward velocity from 0.02 to 0.08 where we find that, at lower initial velocities, the experimental trends narrowly overlaps with the feasible region (see Supplementary information I). As the initial velocity increases, the size of the overlapping region also increases. The maximum overlap is at the nondimensional initial velocity of 0.04. Using the relationship between maximum leg length of the ant and the maximum tripod length from the feasible region, we retrieve the length at rest of the tripod, $L_{0}$, as listed in Table 1.

**Table 1:** Expected gait characteristics extracted from the proposed model.

	Body properties			Locomotion kinematics			
Species	Rest leg length, $L_{0}$ (mm)	Natural frequency, $\omega_{0}$ ($rad\;s^{-1}$)	Stiffness, *k* ($N\;m^{-1}$)	Forward speed, *V* ($mm\;s^{-1}$)	Step frequency, *f* (Hz)	Step length, ΔY (mm)	Amplitude, A (mm)
*C. albicans*	3.6	109–387	0.23–2.99	147–495	41–117	3.0–5.1	0.9–1.9
*C. bicolor*	6.7	79–199	0.17–1.14	169–553	30–68	4.4–9.9	1.5–3.6
*C. bombycina*	4.7	94–238	0.17–1.14	141–462	36–81	3.1–6.9	1.0–2.5
*C. fortis*	5.6	86–220	0.06–0.40	158–502	33–75	3.8–8.1	1.2–2.9
*F. polyctena*	3.6	109–137	0.25–0.40	10–24	41–67	1.6–5.5	0.5–1.4

To improve the accuracy of estimating gait metrics from a known metric (such as the forward speed), we construct a constraint region informed by empirical forward speed-step length and forward speed-step frequency trends. Specifically, we begin by identifying data points that lie close to trend lines, and extract the values of stiffness and efficiency. Then, the minimum and maximum bounds for stiffness and efficiency are computed and used to determine two subregions of like points in the feasible region. The intersection of these subregions provide informed bounds on the body and locomotion properties, as well as the various energy metrics (Tables 1 and 2, respectively). By narrowing the feasible region to a smaller set of points, we determine the range for the forward speed at which our model captures the locomotion dynamics and are capable of informing the energetic costs of individuals moving at that speed.

**Table 2:** Energy metrics associated with walking ants.

Species	Efficiency (%)	CoT ($\text{J}\;{\text{kg}}^{-1}\;{\text{m}}^{-1}$)	Input energy, $e_{\mathrm{in}}$ (μJ)	Percentage congruity
*C. albicans*	0.90–1.52	252–3,036	19–252	48.6–50.4
*C. bicolor*	0.71–1.41	256–3,255	50–343	48.7–50.4
*C. bombycina*	0.72–1.45	256–3,255	24–167	48.7–50.4
*C. fortis*	0.77–1.41	256–2,932	12–82	48.7–50.4
*F. polyctena*	0.52–1.92	252–1,090	20–34	49.5–50.4

The model captures well the trends extracted from the experimental data at high speeds, providing a foundation for an accurate input energy estimate. For *C. fortis*, the forward speed range captured by the model ranges from ∼158 to $∼502\;\text{mm}\;{\text{s}}^{-1}$ (Fig. 6a and b and Table 1). In the case of *C. bombycina* ants, the range is similar, with the overlapping region falling between 141 and $462\;\text{mm}\;{\text{s}}^{-1}$ (Fig. 6d and e and Table 1). The likelihood of the tripod gait—borrowed from relative gait frequency trends measured in Pfeffer et al. (29)—is prevalent across speed ranges, where its probability decreases at higher speeds and is species-dependent (Fig. 6a, b, d, and e). We additionally perform analysis on species of *C. bicolor*, *C. albicans*, and *F. polyctena*; data shown in Supplementary informations I and II and Table 1.

With the trend line established, many features of the locomotion may be predicted. As an example, we use the data points aligned with the trend line to compute the corresponding stiffness and efficiency ratio. This is depicted by the constraint region encompassing the allowable range of both parameters—yielding a reasonable range of the internal stiffness of ants with similar morphologies. For *C. fortis* and *C. bombycina*, the efficiency ranges were 0.09−0.40 and 0.09−0.42, respectively, with stiffness ranges 0.0−0.40 and $0.17-1.14\;\text{N}\;{\text{m}}^{-1}$ (Table 1). Finally, to predict the range of allowable φ and *γ*, we translate the overlapping points back to the feasibility region (Fig. 6c and d). The allowable combinations of φ and *γ* are similar in *Cataglyphis* species—satisfying expectations based on their similar morphologies. In an attempt to determine the importance of the vertical oscillations on determining ant energetics, we also compare against model predictions from approximation 8 (Supplementary information I). The model is seldom able to capture either the step frequency or length of the walking dynamics, reinforcing the significance of the vertical dynamics for input energy estimation.

**Figure 7:**
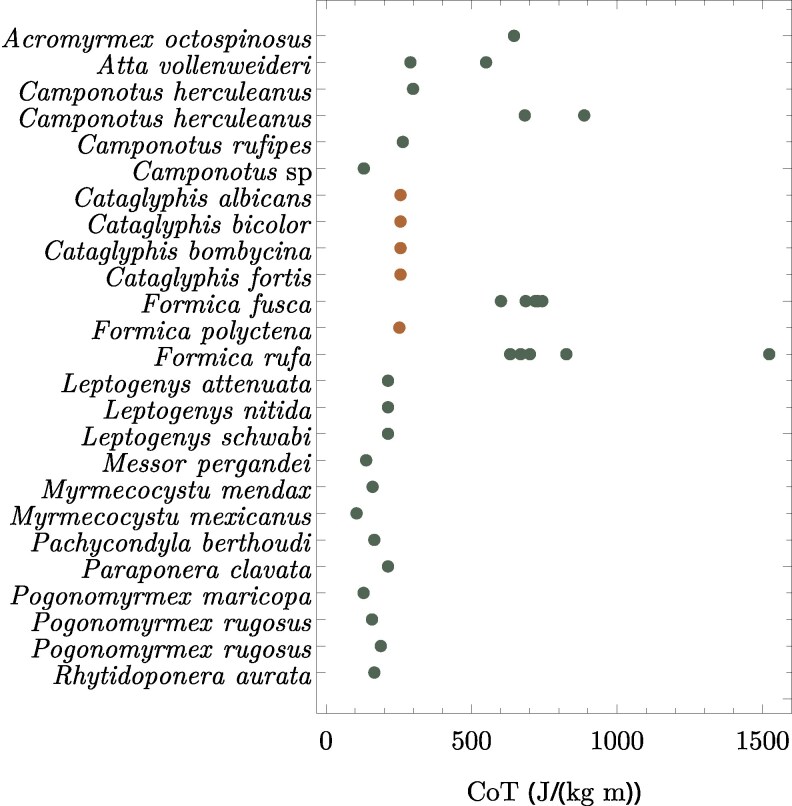
Comparison of the estimated MCoT with values from the literature. A comparative analysis shows that the predicted CoT values for the species examined here (brown)—including *C. albicans*, *C. bicolor, C. bombycina*, *C. fortis*, and *F. polyctena*—are consistent with empirical measurements from the literature (green). Notably, most published values represent MCoT, suggesting the model is capable of capturing low-energy locomotion in insects. Empirical data were extracted from Refs. (6, 7, 10, 60–70). Overlapping data between motion and the energy consumption at the individual-level is currently lacking in the literature—leading to an inability to validate predictions directly.

We list the estimated range of the energy metrics in Table 2, which shows that ant locomotion is characterized by low efficiency and high CoT. The estimated MCoT aligns with the higher values of the published data (Fig. 7). This is likely because our model captures the dynamics at medium to high forward speeds, whereas most published values represent the MCoT—a metric that is independent of forward speed and standard metabolic rate which describes the minimum required energy to sustain life functions. However, our estimates are closely aligned with measurements that are speed-dependent, demonstrating the strong correlation between the model-predicted CoT and experiments. Although flow respirometry has been performed for various ant species, the data on associated CoT is limited in the literature. For this reason, our analysis focuses on species for which motion data is available, even if energetic measurements are lacking. This approach allows us to leverage existing kinematic datasets while acknowledging the current gap in combined biomechanical and metabolic data for desert ants.

## Discussion

Video-based tracking has emerged as a powerful tool for quantifying key behavioral and physiological metrics—including activity patterns, circadian rhythms, and social interactions—that traditionally required special sensors, labor-intensive protocols, or complex experimental setups (17, 18, 21, 22). Motivated by the advances in video tracking that require minimal expertise, we present a unified framework to estimate energy consumption of terrestrial walking insects. We integrate motion tracking with physics-based modeling to estimate the energetics of walking and demonstrate its effectiveness in estimating energy consumption in ants. Using data extracted from video recordings, we estimate kinematic parameters—forward speed, step length, step frequency, and maximum leg length—and use them to map ants’ locomotion onto a feasible locomotion region well-described by a simple walking model. This mapping allows us to infer input energy estimates and explore gait dynamics, morphological adaptations, and trade-offs between adaptability and efficiency.

Our biomechanical analysis highlights the importance of incorporating vertical oscillations to accurately capture the dynamics of ant locomotion, which is often neglected when studying insects whose CoM maintains a persistent height while walking. This reinforces the hypothesis that ants—as hexapods—tend to adopt higher stepping frequencies in response to substrate irregularity, consistent with the grain size hypothesis (71). An analysis of the input energy predicted by the model further reinforces that ants prioritize stability and adaptability, adopting higher step frequencies and maintaining ground contact, even at the cost of energetic efficiency. This is reflected in our model’s prediction of high CoT and low locomotion efficiency. The model effectively differentiates the locomotion dynamics of *Cataglyphis* and *F. polyctena*. While the model captures the locomotion dynamics of *Cataglyphis* species, the forward speed–step relationships of *F. polyctena* falls outside the feasible region. This highlights both the limitations of applying a generalized locomotion model across species and the value of identifying species-specific gait characteristics. We propose that the difference in dynamics is a result of ecological and morphological adaptations that directly influence the gait type and energetic demands. *Cataglyphis*, a thermophilic species of ants, has longer legs adapted for desert climates where they walk faster and with an erect posture (45). In contrast, a nonthermophilic species *F. polyctena*—also known as wood ants—have comparatively shorter legs and a lowered gaster, which favors grounded walking (42).

At slower speeds, many ants shift away from the classic tripod gait, adopting longer double-stance phases and higher duty factors—sometimes up to 65 (29, 42–44). This explains why our model, which assumes a 50 duty factor, is best suited for intermediate to high speed locomotion. This limitation may be addressed by adopting alternative actuation profiles that account for variable duty factors across gaits. Accurate input energy estimation, therefore, requires attention to gait variability across species and speeds. One may also consider the effects of inclined surfaces on the trajectory of the CoM and energy consumption. Lipp et al. (10), for instance, find that ants walking on inclined surfaces maintain a consistent forward speed while experiencing a higher metabolic cost. The model presented here may be adapted to inclined surfaces by applying gravity along a force vector which deviates from the position normal to the walking surface, and according to the angle of the incline. In this case, we should expect changes in the feasible walking region to account for the inability of the ant to maintain stable walking on inclines of increasing magnitude.

Additionally, our analysis aligns with the hypothesis that relative leg stiffness is conserved across animals, regardless of morphology or gait (72). By using a fixed body mass and leg length across individuals, we extract a range of relative stiffness values that align with previous findings (Supplementary information II) (72). This consistency underscores the generalization of stiffness-based modeling between insect species. Our CoT analysis further supports the notion that locomotion energetics reflect species-specific morphological and ecological adaptations (57, 73–75). For instance, ants’ high step frequency and short step length, linked to their small size, drive their elevated metabolic cost, placing them at the top of the metabolic cost hierarchy (7, 76, 77). Furthermore, higher step frequency and shorter step length require the recruitment of faster-muscle fibers that consume more energy (57). This expectation parallels that of Kleiber’s law, where metabolic cost scales with a $\frac{3}{4}$ power of body mass, implying lower efficiency in smaller animals (78). We validate our estimated CoT by comparing it with available data on ant species. However, the absence of species for which both kinematic and CoT data are simultaneously available limits our ability to draw direct comparisons. Despite this limitation, our estimates for the lower limits of the CoT for walking ants align closely with all measured values considered from the literature, suggesting that with more granular tuning of the various model parameters we will be able to capture the energetics of individuals.

Despite individual ants exhibiting low locomotion efficiency and high CoT compared to mammals, birds, reptiles, amphibians, crustaceans, and myriapods (76), this apparent inefficiency at the individual level may be reconciled by colony-level energetic efficiency. Our findings may help explain why colonies maintain overall energetic efficiency. Collective behaviors—such as task allocation and the suppression of redundant activity—enable colonies to reduce total energy expenditure as they grow (79, 80). Through such strategies, energy expenditure scales sublinearly with colony size—enabling the colony (a “superorganism”) to optimize overall efficiency, not via individual biomechanics, but through emergent organization (81, 82). This paradigm reconciles two seemingly contradictory observations: (i) our model’s prediction of high individual energy costs during locomotion and (ii) empirical measurements of colony-level metabolic efficiency. Social coordination may emerge as an adaptive response to the constraints of inefficient individual locomotion, allowing colonies to thrive in heterogeneous environments while conserving energy at scale.

A limitation of the current model is its reliance on a fixed duty factor, which reduces its ability to capture ants that utilize double stance, or aerial phases, while walking and running. Similarly, assumptions on the ground contact limit the model’s ability to pick up on more detailed contact mechanics, such as surface adhesion or substrate friction. Our initial study shows that internal damping plays a secondary role on the energetics of locomotion (Supplementary information I); future work should further explore the role of damping. Currently, the CoM dynamics are considered only at the steady state, leaving room for improvement in estimating the individual’s transient energetic consumption. The mathematical framework can be complemented with a lateral degree of freedom to investigate the role of lateral oscillations in stability, adaptability, and energy consumption. While the proposed constraint region refines the estimation of gait metrics using experimental forward speed-step length and forward speed-step frequency trends, it is limited to the specified species. In future work, one should validate the model’s robustness across a wider range of insect species. Enhancing the locomotion model—for instance, by incorporating gait variability and transient dynamics—could increase accuracy at lower speeds and broaden its applicability. We also aim to adapt this video-based methodology to estimate energy use in other social insects, an approach not yet implemented in this context. Such studies could illuminate how individual energy costs influence colony-level energetics, offering new insights into the metabolic strategies of eusocial organisms. Finally, our approach for energy estimation assumes fixed values for the actuation amplitude, step size, and initial forward velocity; measuring these parameters as well could further improve the accuracy of the model’s estimates of energy consumption.

Our work provides an effective, simple alternative to estimate the energetics of locomotion in terrestrial insects like ants. The method captures biologically relevant changes in CoT influenced by environmental conditions such as temperature and humidity, as reflected in gait adjustments seen in the video data (9, 10). It accurately captures the input energy of walking ants, reflecting the validity of video-based energy estimation. The proposed framework, with multiple runs, constructs the speed-dependent trends when overlapped on the feasible region, giving important insights into fundamental biomechanical principles—for example, stiffness-frequency trade-offs, adaptability–efficiency balances, colony-level optimization, and gait transition dynamics. This approach lays the groundwork for future investigations into video-based energy estimation in insects. This approach lays the groundwork for future investigations into video-based energy estimation in insects and provides insight for the design of energy-efficient robotic systems (37, 38).

## Methods

### Numerical simulations

When simulating the nondimensional model, we step when the nondimensional form of [4] is satisfied within a strict numerical threshold, $\varepsilon =10^{-16}$ (Supplementary information I). This condition is expected to allow the body to adjust its height and vertical velocity such that at steady state, one should recover periodicity in the vertical motion as well, namely,


(7) $$z(\tau_{n}+\Delta \tau )=z(\tau_{n}),\;\dot{z}(\tau_{n}+\Delta \tau )=\dot{z}(\tau_{n}).$$

Our expectation of periodicity in the vertical coordinate is based on the coupling between the vertical and forward dynamics in [1]. We consider that a limit cycle is reached by checking [7] for successive events—stopping when the absolute differences in both *z* and $\dot{z}$ are within $10^{-4}$.

### Simplified model

We neglect the effect of the acceleration of gravity on the walking trajectory and write the model only on the forward axis. Time is scaled similar to [6], while the length is instead scaled by $L_{d}^{\prime }$, which is the amplitude of the actuation function. The equation of motion reduces to


(8) $$\ddot{y}(\tau )=\varphi^{2}(\ell_{\mathrm{eq}}^{\prime }(\tau )-\ell_{\mathrm{tri}}^{\prime }(\tau )),$$

where we use a prime to differentiate between variables in the sagittal plane and their projection on the forward axis. The actuation function is $\ell_{\mathrm{eq}}^{\prime }(\tau )=\ell_{0}^{\prime }-\cos\;(\tau )$, and the tripod length simplifies to $\ell_{\mathrm{tri}}^{\prime }(\tau )=y(\tau )-y_{f}$, yielding a linear, second-order differential equation. We are left with only three independent variables, namely, φ, $\dot{y}(0)$, and $\ell_{0}^{\prime }$.

### Determination of a feasible region

To estimate the length of the leg at rest from data—the quantity we use as the common length scale—we start from $L_{\max}$, which is generally available from the literature. From the feasible region, we infer $\ell_{\max}$ and then estimate $L_{0}$ as $L_{0}=\frac{L_{\max}}{\ell_{\max}}$. Finally, the stiffness coefficient is computed from *γ* as $k=m(\varphi \omega {)}^{2}$, using the ant’s mass and actuation frequency, with φ taken from the feasible region. To evaluate the stability of a walking trajectory within the feasible region, we construct a Poincaré section defined by the hyperplane $\dot{y}(0)=\dot{y}(\tau )$. We consider a single window at the end of the simulation as representative of the steady state dynamics. Treating the resulting Poincaré map as a discrete dynamical system, we quantify the stability of the walking trajectory by numerically solving for the eigenvalues (Floquet multipliers) corresponding to small perturbations in the trajectory at the beginning of the step window (83). The eigenvalues characterize the growth or decay of trajectories through the Poincaré map and across adjacent events. For the system to be stable, the spectral radius (the highest magnitude of the three Floquet multipliers) must be <1. We find that for each point in the feasible region, the spectral radius is <1—implying a stable periodic trajectory (Supplementary information I).

### Energy metrics

We consider a single steady-state gait window to be representative of the energy dynamics over the full range of motion. Within this gait cycle, we first estimate the instantaneous, nondimensional ground reaction force (GRF) transmitted through the leg, defined as


(9) $$f_{\mathrm{leg}}(\tau )=\varphi^{2}(\ell_{\mathrm{eq}}(\tau )-\ell_{\mathrm{tri}}(\tau )).$$

The nondimensional energy consumed over a gait window, or input energy, is proportional to the integral of the absolute value of the GRF multiplied by the rate of change in the actuation length (84),


(10) $$e_{\mathrm{in}}=\int_{0}^{\Delta \tau }|f_{\mathrm{leg}}(\tau ){\dot{\ell }}_{\mathrm{eq}}(\tau )|\text{d}\tau .$$

With this, we compute the CoT and the efficiency of the gait as simple functions of $e_{\mathrm{in}}$. The CoT is the energy utilized to move the ant mass a unit distance, that is,


(11) $$\text{CoT}=\frac{e_{\mathrm{in}}}{\Delta y}.$$

The gait efficiency is defined as the part of the input energy converted into productive motion, or the forward locomotion of the CoM, compared to the input energy. We estimate efficiency by taking the average velocity over a gait cycle, $v=\frac{\Delta y}{\Delta \tau }$ (in dimensional form, *V*), and using it to compare the forward kinetic energy to the energy inputted into the system, namely,


(12) $$\%\text{eff}=\frac{v^{2}}{2e_{\mathrm{in}}}100\%.$$

To bring the energetic quantities to physically relevant units, we multiply the energy input by $mL_{0}^{2}\omega^{2}$ and CoT by $L_{0}\omega^{2}$, and adjust the step and cycle length as discussed in “Determination of a feasible region” section.

Necessary for validating the model on existing data, we also require the percentage congruity of the gait cycle, or the proportion of time the kinetic and potential energies (ke and pe, respectively) are in phase. We compute the percentage congruity from the nondimensional trajectory as


(13) $$\text{\textbackslash{}\% Congruity}=\left(\frac{1}{\Delta \tau }\int_{0}^{\Delta \tau }I\left[\frac{\text{d}}{\text{d}\tau }\text{ke}(\tau )\cdot \frac{\text{d}}{\text{d}\tau }\text{pe}(\tau )>0\right]\text{d}\tau \right),$$

where I is an indicator function which yields 1 when the condition holds and 0 otherwise, and the quantity is made into a percent when reported. The kinetic energy used here encompasses both the forward and vertical directions, $\text{ke}=\frac{1}{2}\sqrt{{\dot{y}}^{2}(\tau )+{\dot{z}}^{2}(\tau )}$ and pe=γz(τ).

## Supplementary Material

pgag174_Supplementary_Data

## Data Availability

All code needed to reproduce the results of this article are provided on Github (85).
