# Supplementary material for: A model-based approach to study ant energetics from trajectory data
Source: PNAS Nexus. 2026 May 28;5(6):pgag174. doi: 10.1093/pnasnexus/pgag174 (PMC13255996; doi:10.1093/pnasnexus/pgag174)
Supplement: pgag174_Supplementary_Data [file pgag174_supplementary_data.zip › PNASNEXUS-PNASNEXUS-2025-01520R-s01.pdf]

Supplementary Information

A model-based approach to study ant energetics from trajectory data

Basit Yaqoob[1,2,3], Michael Napoli[1,2], Nicola Pugno[4,5], and Maurizio Porfiri*[1,2,6,7]

[1]Center for Urban Science and Progress, Tandon School of Engineering, New York University, Brooklyn, New York, 11201, USA

[2]Department of Mechanical and Aerospace Engineering, Tandon School of Engineering, New York University, Brooklyn, New York, 11201, USA

[3]Department of Mechanical Engineering, National University of Technology, Sector I-12, Islamabad, Pakistan

[4]Mechano-X Labs, Department of Civil, Environmental and Mechanical Engineering, University of Trento, Trento 38122, Italy

[5]School of Engineering and Materials Science, Queen Mary University of London, London E1 4NS, UK

[6]Department of Civil, Urban, and Environmental Engineering, Tandon School of Engineering, New York University, Brooklyn, New York, 11201, USA

[7]Department of Biomedical Engineering, Tandon School of Engineering, New York University, Brooklyn, New York, 11201, USA

1 Supplementary Results on the Mathematical Model

1.1 Trajectories

An example of a complete trajectory of the center of mass (CoM) is provided to show the transition period (Figure S1; Supplementary Video 1). Note that depending upon the actuation ratio and inverse Froude number, the transient phase, defined as the number of steps required to reach the steady state, can become longer or shorter.

1.2 Sensitivity Analysis

We conduct a numerical analysis to assess how initial conditions and actuation amplitude influence gait metrics (Figures S2 and S3). Our results demonstrate that the actuation amplitude regulates the amplitude of the vertical oscillations of the CoM, the step length, the forward speed, and the input energy (Figures S2d and S3d), while the initial forward velocity plays an important role mostly on forward speed and step length (Figure S2c). Using the linear relationship between the CoM amplitude and the actuation amplitude (Figure S2d), we bound ℓ_{d} so that the CoM amplitude is less than 2 mm (Figure S4a) – a physiologically realistic value [5]. Similarly, by using the linear relationship between the initial forward velocity and the step length (Figure S2c), we identify a region where plausible values of the initial forward velocity maintain the step length under 20 mm (Figure S4b) – a physiologically realistic value [6, 2, 8, 9, 1, 11, 3]. A similar analysis can be performed for other insects species.

1.3 Stability

To assess the stability of walking trajectories in the feasible region numerically, we use a Poincaré section map [7]. Stability is analyzed by computing the Floquet multipliers, which are the absolute values of the eigenvalues of the linearized return map around the periodic orbit. Mapping the maximum of three Floquet multipliers in the feasible region confirms the stability of the walking gait (Figure S5).

*Corresponding author: mporfiri@nyu.edu

1.4 Gait Metrics in the Feasible Region

We study how the step length, forward speed, mean height of the CoM, amplitude of the CoM vertical oscillations, and convergence steps vary with the non-dimensional parameters φ and γ. Each metric is depicted as a heatmap over the feasible region in Figure S6. The step length and speed of the walking increase proportionally to the frequency ratio φ and the inverse Froude number γ (Figures S6a and b, respectively). The mean height of the CoM (Figure S6c) of the CoM increases with φ and γ. Interestingly, while an increase in γ reduces the mean CoM height, it does not affect the amplitude of the CoM vertical oscillations (Figure S6d). This suggests that the body compensates for a stronger gravitational influence by lowering its baseline posture, while maintaining consistent oscillatory motion. Increasing φ not only elevates the mean CoM height, but also increases the oscillation amplitude of the CoM vertical oscillations, indicating that stiffer systems both lift and amplify vertical motion. The convergence steps are defined as the number of steps required to bring variation between successive steps under 10^{-4}. The system takes longer to converge for extreme values of φ (Figure S6e). The ground reaction force (GRF) increases with φ and γ, suggesting lower values lead to decreased GRF (Figure S6f).

1.5 Influence of the Initial Forward Velocity

We vary the initial forward velocity to find the best overlap between experimental trends from Pfeffer et al. [4] and the feasible region. A non-dimensional initial forward velocity of 0.04 is selected to represent a broad spectrum of locomotor dynamics, as indicated by the highest overlap in Figures S7 and S8. A lower initial forward velocity results in a limited overlap with the feasible region, and a similar effect is observed at higher initial velocities. The locomotion dynamics of *Formica polyctena* differ significantly from the dynamics of *Cataglyphis*; for this reason, the model over predicts the frequency and under predicts the step length, highlighting the significance of the double stance phase for *F. polyctena*.

1.6 Trends of the CoT and Energy Efficiency Percentage in the Constraint Region

We depict the CoT and energy efficiency over the constraint region constructed in Section 2.3 of the main text. The CoT, perhaps trivially, increases as a function both of the speed of travel, V, and the step length, ΔY, when the ant is moving at a high-speed (Figure S9a, c, e, and g), which is most relevant in the *Cataglyphis* genus considered here. When the ant moves slowly, however, we observe an opposite trend (Figure S9i; *F. polyctena*), suggesting the existence of a potentially "optimal" walking speed. The energy efficiency follows a similar trend, with efficiency increasing as a function of the step length in all but *C. albican* (Figure S9b), suggesting that longer steps benefit the efficiency of the walking trajectory (Figure S9d, f, h, and j).

2 Supplementary Results on the Simplified Model

The forward position and velocity of the simplified model are shown in Figure S10a and b, respectively. Our implementation of the model is characterized by large changes in the forward velocity $\dot{y}$ which repeat continuously. The two stance phases are synonymous with one another and alternate continuously. Unlike the complete model, the dynamics in the ground plane do not contain a transient phase.

2.1 Dynamics in the Ground Plane Assumption

To understand how well the ground plane assumption captures the dynamics of ant locomotion, we vary the initial forward velocity and assess the accuracy of the model in terms of the degree of overlap between the experimental speed-step length and frequency trends from the literature [4, 5, 10] and the predictions extracted from the model. We find that the simplified model consistently underestimates the step length of the ant species, underperforming in comparison to the complete model and highlighting the importance of the vertical dynamics (Figures S11 and S12).

3 Supplementary Results on the Influence of Damping

To incorporate the influence of a dissipative force on the CoM walking trajectory, we include a damping term that oscillates in-phase with the equilibrium length L_{eq} and whose strength is parameterized by the damping coefficient b. The dimensional form of the damped walking model is written

$$m\ddot{Y}(t) = (-k(L_{\text{tri},n}(t) - L_{\text{eq},n}(t)) - b(\dot{L}_{\text{tri},n}(t) - \dot{L}_{\text{eq},n}(t)))\frac{Y(t) - Y_{\text{f},n}}{L_{\text{tri},n}(t)}, \tag{1a}$$

$$m\ddot{Z}(t) = (-k(L_{\text{tri},n}(t) - L_{\text{eq},n}(t)) - b(\dot{L}_{\text{tri},n}(t) - \dot{L}_{\text{eq},n}(t)))\frac{Z(t)}{L_{\text{tri},n}(t)} - mg, \tag{1b}$$

We non-dimensionalize (1) using the same methods as described in the main text, that is, using a length scale proportional to L_0 and time scale proportional to ω. For simplicity we omit the index of the gait window, allowing us to write

$$\ddot{y}(\tau) = (-\varphi^2(\ell_{\text{tri}}(\tau) - \ell_{\text{eq}}(t)) - 2\zeta\varphi(\dot{\ell}_{\text{tri}}(\tau) - \dot{\ell}_{\text{eq}}(\tau)))\frac{y(\tau) - y_{\text{f}}}{\ell_{\text{tri}}(\tau)}, \tag{2a}$$

$$\ddot{z}(\tau) = (-\varphi^2(\ell_{\text{tri}}(\tau) - \ell_{\text{eq}}(\tau)) - 2\zeta\varphi(\dot{\ell}_{\text{tri}}(\tau) - \dot{\ell}_{\text{eq}}(\tau)))\frac{z(\tau)}{\ell_{\text{tri}}(\tau)} - \gamma, \tag{2b}$$

where $\zeta = \frac{b}{2\sqrt{km}}$ is the non-dimensional damping ratio. We likewise adjust the equation for the non-dimensional force exerted through the leg, f_{leg}, reported in the main text such that

$$f_{\text{leg}} = \varphi^2(\ell_{\text{eq}}(\tau) - \ell_{\text{tri}}(\tau)) + 2\zeta\varphi(\dot{\ell}_{\text{eq}}(\tau) - \dot{\ell}_{\text{tri}}(\tau)). \tag{3}$$

We test the damped walking model for each of the energetic and walking trends tested in the main text, finding that the incorporation of a biological feasible value for the damping ratio ($\zeta = 0.05$) does not significantly change the results of the undamped model (Figure S13). Intuitively, we measure increases in the CoT and input energy and a decrease in the energy efficient for like-combinations of φ and γ (Figure S13a-c). The step length decreases with the incorporation of damping (Figure S13d). While the walking speed and mean height (Figure S13e and f, respectively) remain consistent with the undamped model results, the body posture (as indicated by the amplitude; Figure S13g) increases, implying that the assumption that vertical oscillations dominate the walking dynamics becomes more accurate with the incorporation of damping. Finally, and perhaps most importantly, the percentage congruity increases with the incorporation of damping (Figure S13h). This finding suggests that variations in the percentage congruity are modulated by the internal damping, thereby calling for future research that may use this parameter to achieve the experimentally measured congruity.

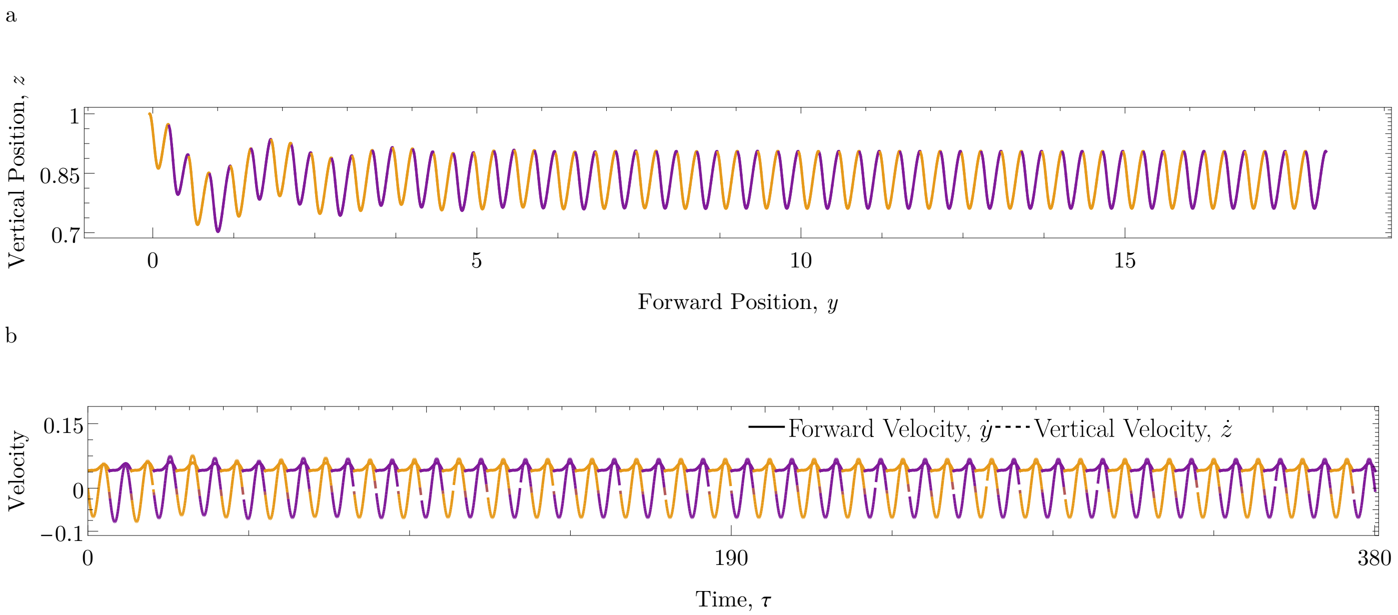

Figure S1: Complete walking trajectory of the CoM until desired convergence is achieved. Parameters and initial conditions are $\varphi = 0.204$, $\gamma = 0.0041$, $z_0 = 1$, $\dot{z}_0 = 0$, $\dot{y}_0 = 0.04$, $\ell_\mathrm{d} = 1.5$, and $\delta y = 0.05$. Changing colors represent switching between alternating tripods.

Supplementary Video 1 (separate file): The evolution of the tripod gait during steady-state locomotion after removing the initial transition of the dynamics. The model is simulated for non-dimensional parameters $(\varphi, \gamma, \ell_\mathrm{d}, \delta y) = (0.0041, 0.204, 1.5, 0.05)$ and initial conditions $(z_0, \dot{z}_0, \dot{y}_0) = (1, 0, 0.04)$. The time evolution of the tripod and the CoM is illustrated for approximately 80 non-dimensional time steps. Alternating colors represent the transition of the active tripod between steps, allowing for a clear differentiation between successive gait windows.

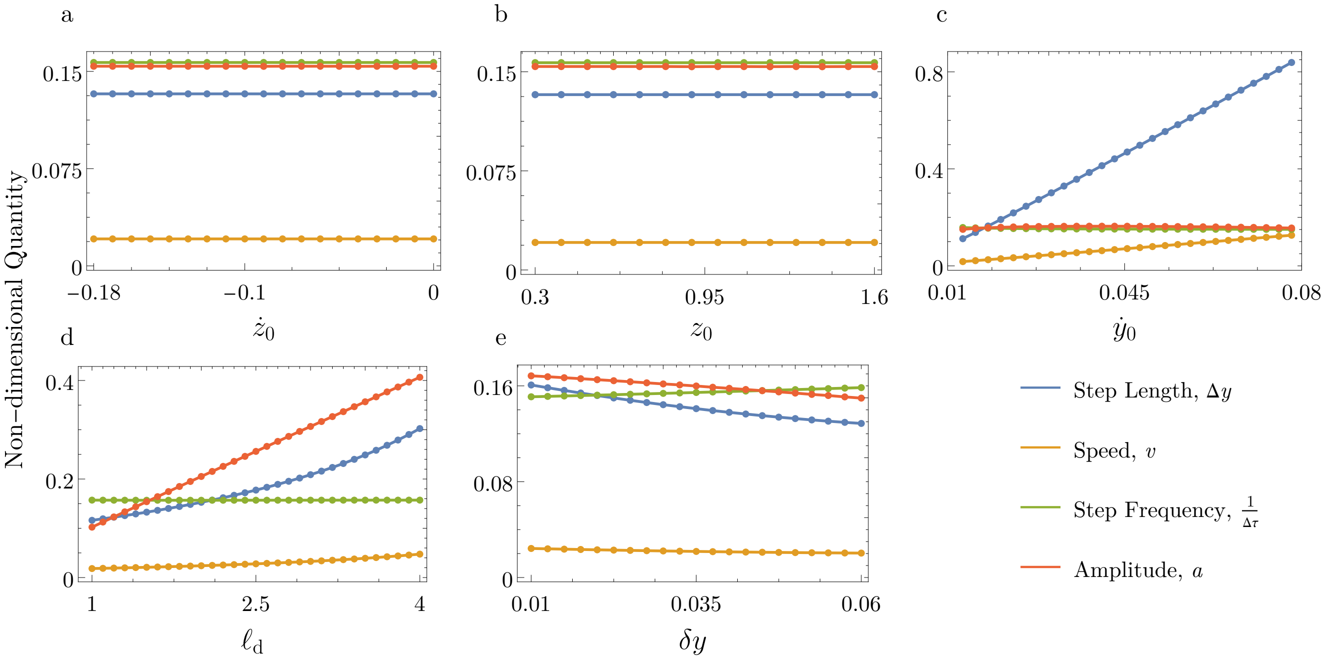

Figure S2: Influence of initial conditions and parameters on gait characteristics (step length, speed, step frequency and amplitude). Simulations are run for $\varphi = 0.3$, $\gamma = 0.01$, $z_0 = 1$, $\dot{z}_0 = 0$, $\dot{y}_0 = 0.015$, $\ell_\mathrm{d} = 1.5$, and $\delta y = 0.05$ unless otherwise specified in the panel. (a) Gait metrics are approximately independent of the initial vertical velocity, $\dot{z}_0$. (b) Gait metrics are nearly independent of the initial height, z_0. (c) The initial forward velocity regulates step length, Δy: the linear relationship between the initial forward velocity and the step length is $\Delta y = 11.22\dot{y}_0 - 0.039$. The initial forward velocity also has an effect on the forward speed. (d) Increasing the actuation amplitude raises the CoM amplitude and step length: the linear relationship between the actuation amplitude and the CoM amplitude is defined by $a = 0.10\ell_\mathrm{d} + 0.0019$. The actuation amplitude also affects the forward speed and the step length. (e) Gait metrics are largely independent of the step size, δy.

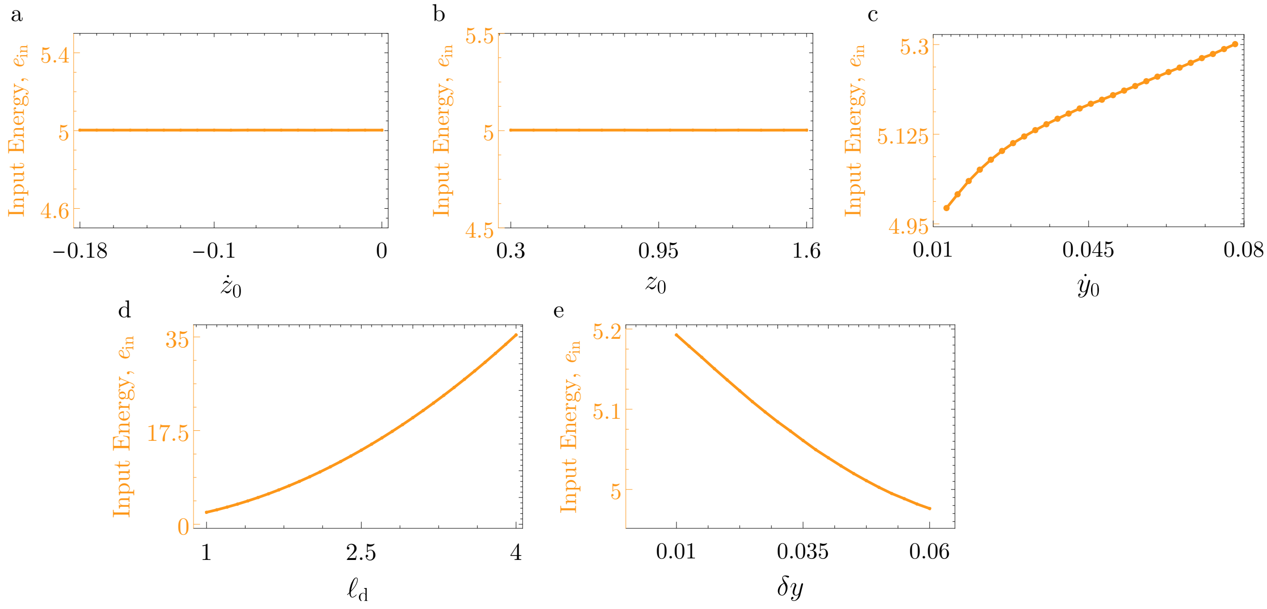

Figure S3: Influence of initial conditions and model parameters on input energy. System parameters and initial conditions are same as of Figure S2. Influence of initial downward velocity (a), initial height (b), initial forward velocity (c), actuation amplitude (d), and step size (e). Input energy is strongly influenced by actuation amplitude, followed by slight variations due to change in δy and $\dot{y}_0$.

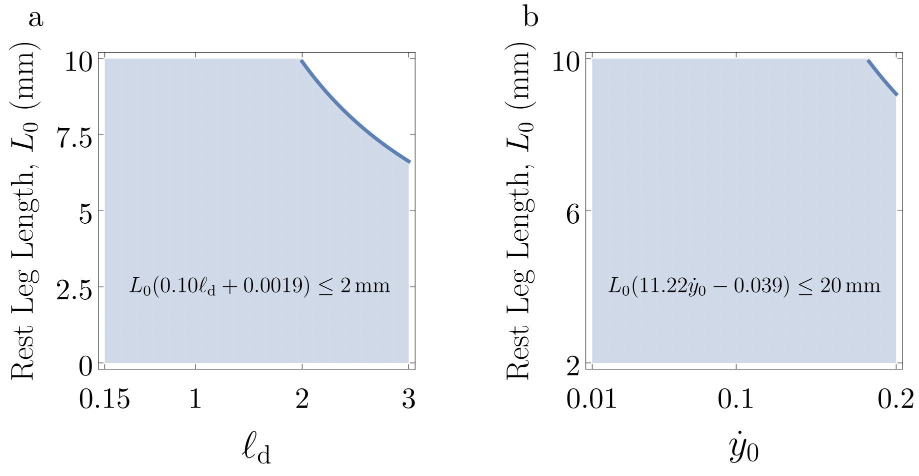

Figure S4: Region where desired constraints on gait kinematics are satisfied. (a) The relationship between actuation amplitude and the CoM amplitude (from Figure S2) is exploited to construct a region in which the CoM amplitude does not exceeds 2 mm. A Higher rest length requires lower actuation amplitude to maintain the CoM amplitude below 2 mm. (b) The initial forward velocity has strong influence on the step length so that we exploit the linear relationship between initial forward velocity and step length to construct a region where the step length does not exceeds 20 mm.

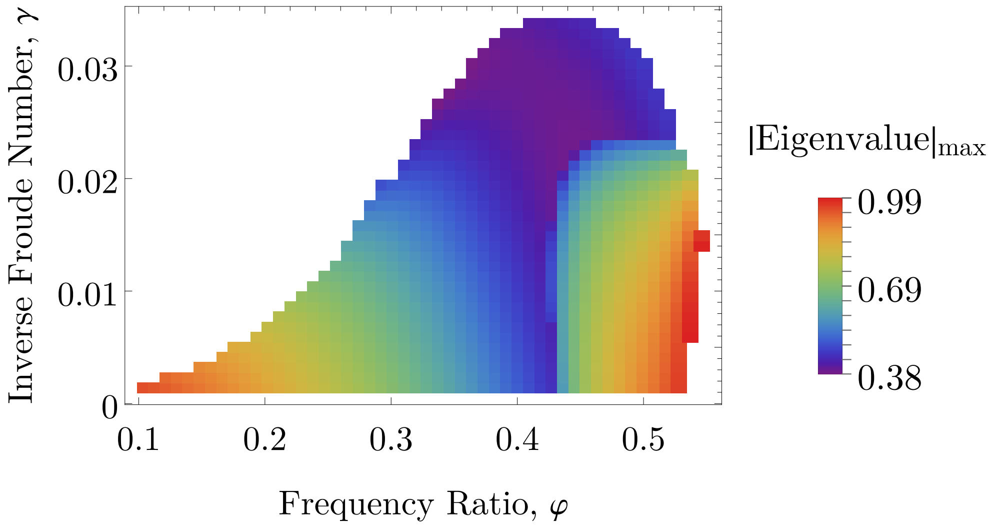

Figure S5: All the trajectories in the feasible region are stable. The periodic gait is perturbed to find the Floquet multipliers. The maximum value of the calculated Floquet multipliers is shown as the heat map, which is always below one – indicating stability of the walking trajectories.

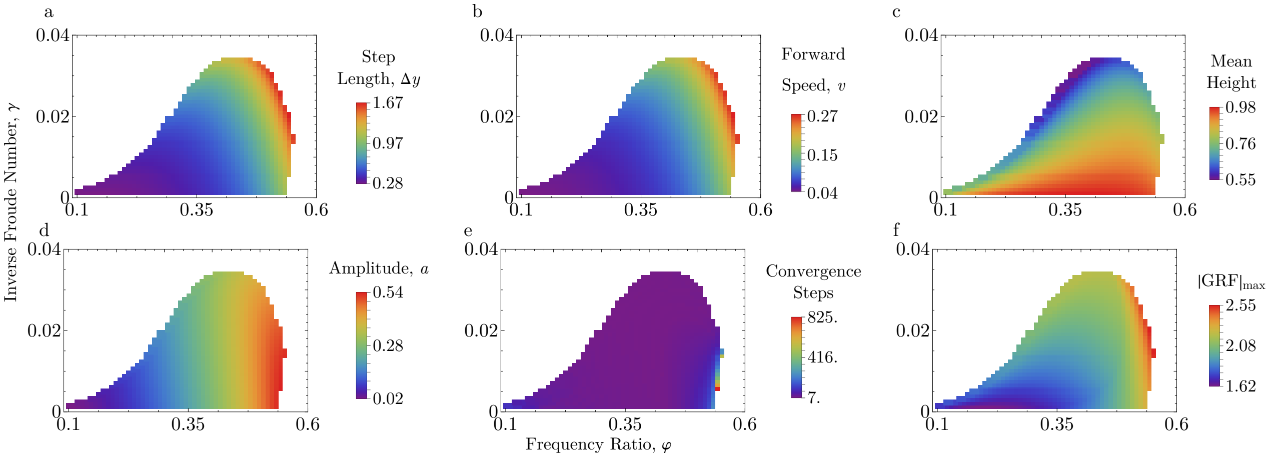

Figure S6: Non-dimensional gait metrics plotted over the feasibility region as functions of parameters φ and γ. Parameters and initial conditions are $z_0 = 1$, $\dot{z}_0 = 0$, $\dot{y}_0 = 0.04$, $\ell_\text{d} = 1.5$, and $\delta y = 0.05$. Step length (a) and forward speed (b) correlate strongly with one another, and they increase along both the φ- and γ-axes. The mean height of the walking trajectory (c) decreases dramatically with γ, and more subtly with φ. The amplitude of the vertical oscillations of the CoM (d) increases proportionally with φ, but remains relatively constant with respect to γ. (e) As the frequency ratio increases, the number of steps needed to achieve steady state increases. (f) Minimizing the ground reaction force requires minimizing the two dimensionless numbers.

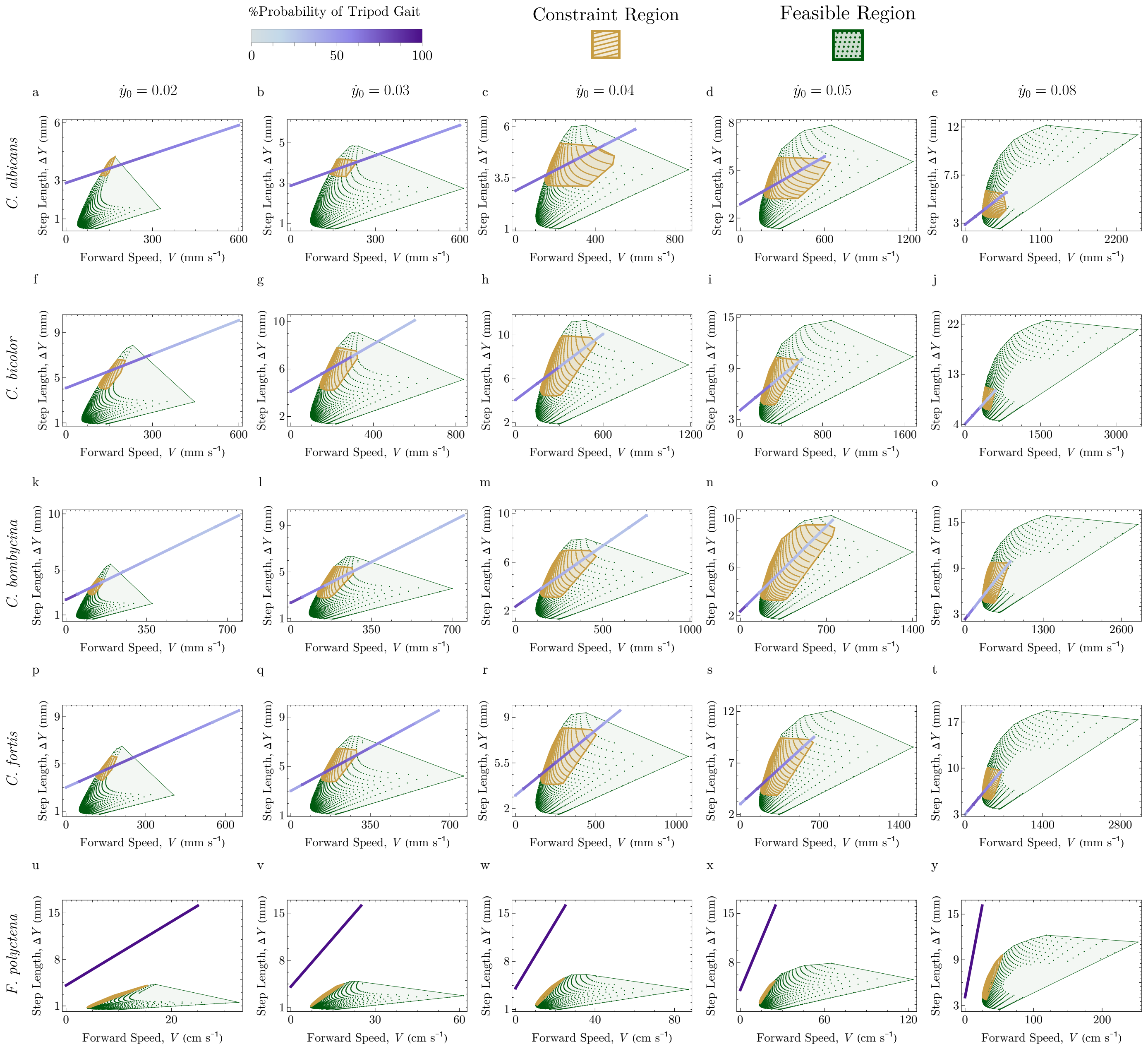

Figure S7: Varying the initial forward velocity to determine the best overlap between the feasible region and trends between step length and forward speed from the literature [4, 5, 10]. Species include: *C. albicans* (a-e), *C. bicolor* (f-j), *C. bombycina* (k-o), *C. fortis* (p-t), and *F. polyctena* (u-y). Parameters and initial conditions are $z_0 = 1$, $\dot{z}_0 = 0$, $\ell_\mathrm{d} = 1.5$, and $\delta y = 0.05$. At each point in the feasible region, we compute the forward speed and step length corresponding to that (φ, γ)-pair. Then, using maximum leg lengths, L_max, of 5.36 mm, 10 mm, 7 mm, 8.25 mm, and 5.39 mm for *C. albicans* (a-e), *C. bicolor* (f-j), *C. bombycina* (k-o), *C. fortis* (p-t) and *F. polyctena* (u-y), respectively, we rescale each point in the non-dimensional region to the forward speed versus step length domain. An initial velocity, $\dot{y}_0$, of 0.04 captures the majority of the experimental trends between forward speed and step length. Increasing the initial velocity shifts the model's validity range toward higher forward speeds and reduces the overlap. Similarly, decreasing the initial velocity shifts the model's validity towards lower forward speeds and reduces the overlap.

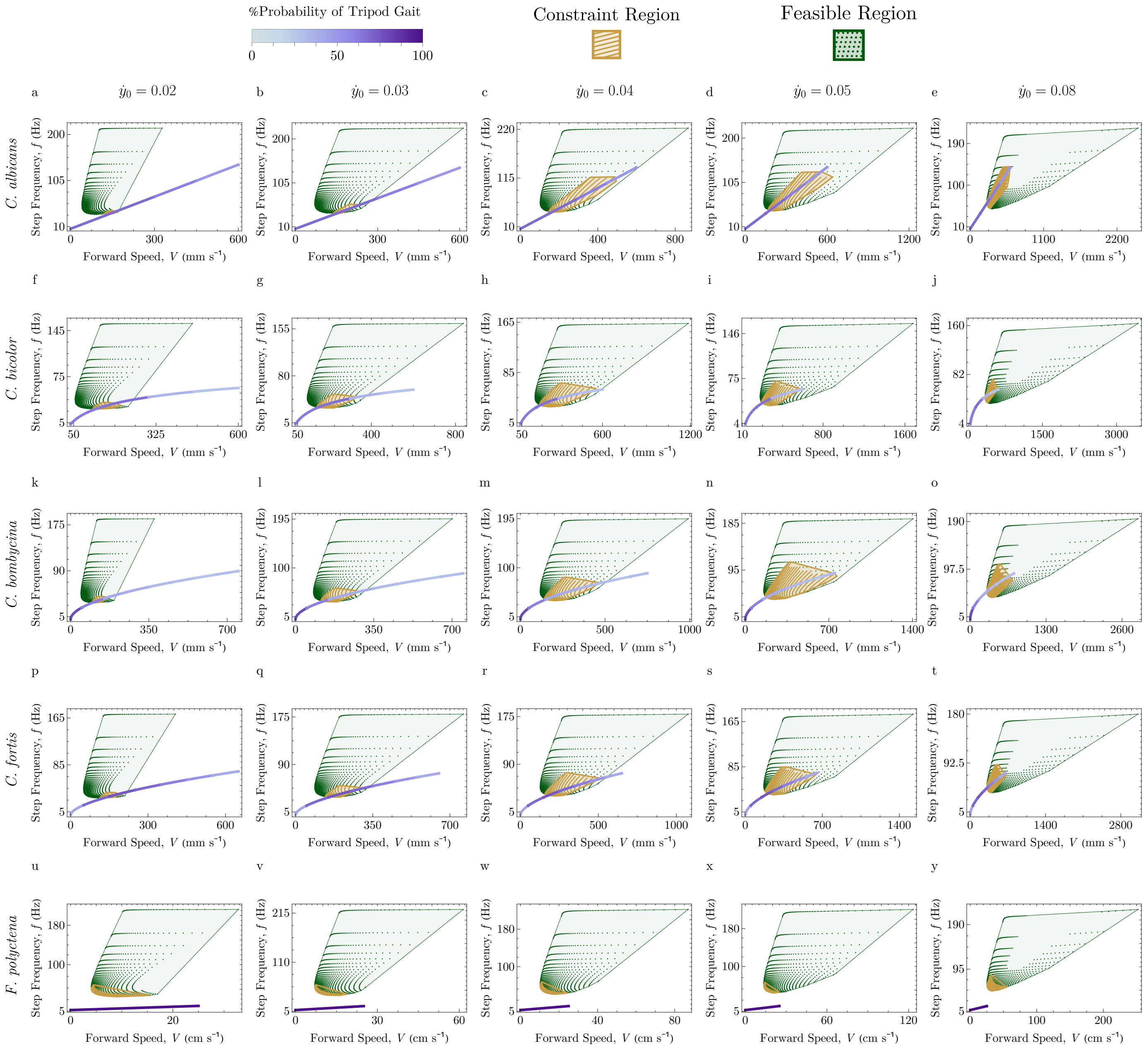

Figure S8: Varying the initial forward velocity to determine the best overlap between the feasible region and trends between step frequency and forward speed from the literature [4, 5, 10]. Species include: *C. albicans* (a-e), *C. bicolor* (f-j), *C. bombycina* (k-o), *C. fortis* (p-t), and *F. polyctena* (u-y). Parameters and initial conditions are $z_0 = 1$, $\dot{z}_0 = 0$, $\ell_{\mathrm{d}} = 1.5$, and $\delta y = 0.05$. At each point in the feasible region, we compute the speed and step frequency corresponding to that (φ, γ)-pair. Then, using maximum leg lengths, $L_{\max}$, of 5.36 mm, 10 mm, 7 mm, 8.25 mm, and 5.39 mm for *C. albicans* (a-e), *C. bicolor* (f-j), *C. bombycina* (k-o), *C. fortis* (p-t) and *F. polyctena* (u-y), respectively, we rescale each point in the non-dimensional region to the forward speed versus step frequency domain. An initial forward velocity, $\dot{y}_0$, of 0.04 captures the majority of the experimental trends between forward speed and step frequency. Increasing the initial velocity shifts the model's validity range toward higher forward speeds and reduces the overlap. Similarly, decreasing the initial velocity shifts the model's validity towards lower forward speeds and reduces the overlap.

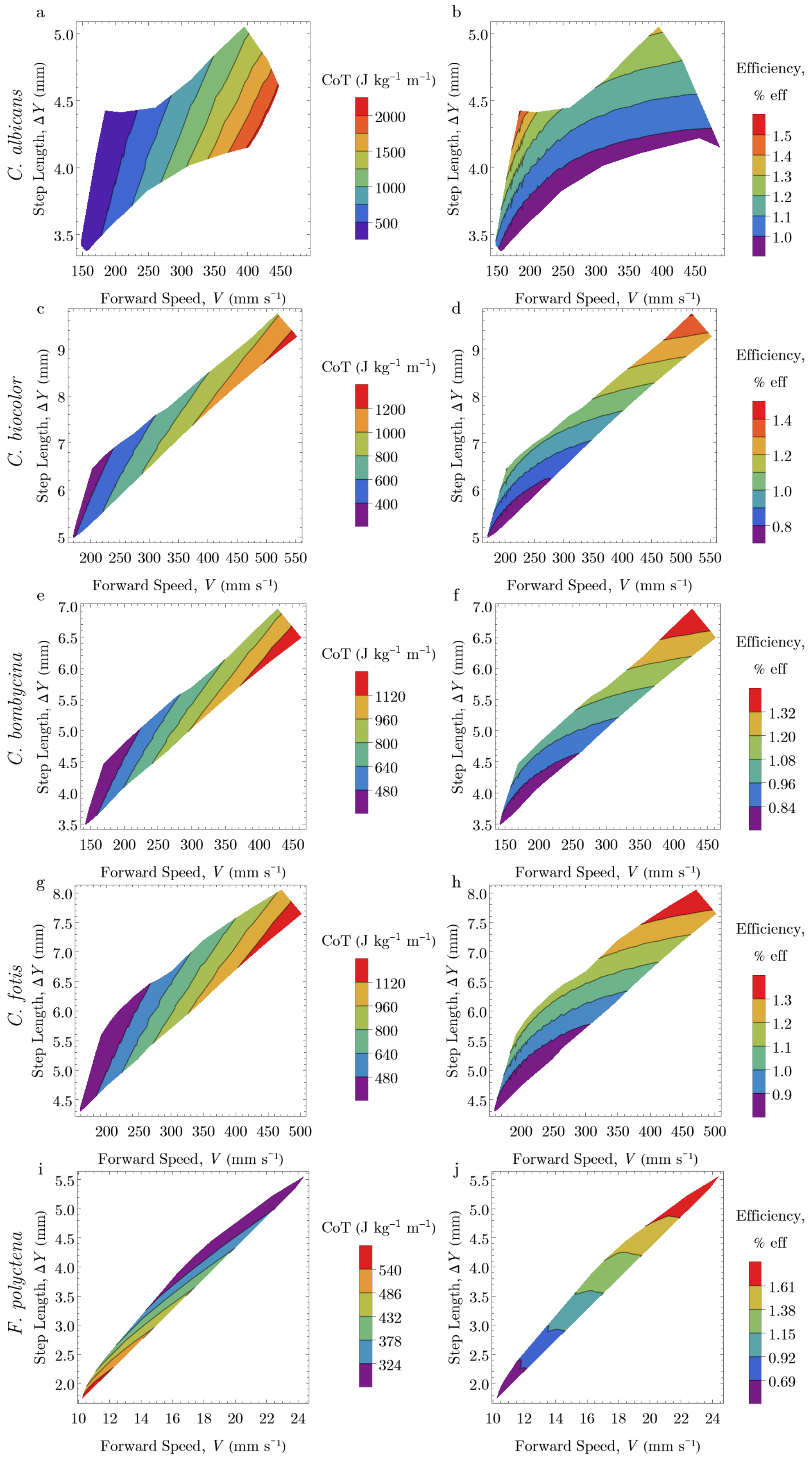

Figure S9: Heat maps depicting the CoT and energy efficiency in the constrained region reported in Figure 6 of the main text. The CoT, perhaps trivially, increases as a function of both the forward speed, V, and the step length, ΔY, in fast-moving ants (a, c, e, and g); respectively, *C. albicans*, *C. bicolor*, *C. bombycina*, *C. fortis*). When the ant moves slowly, however, we observe an inverted trend (i; *F. polyctena*), whereby the CoT benefits from an increases in the speed and step length. This suggests the potential for an "optimal" speed in the model. Likewise, the energy efficiency increases as a function of the forward speed and step length in all species (d, f, h, and j) except that of *C. albican* (b).

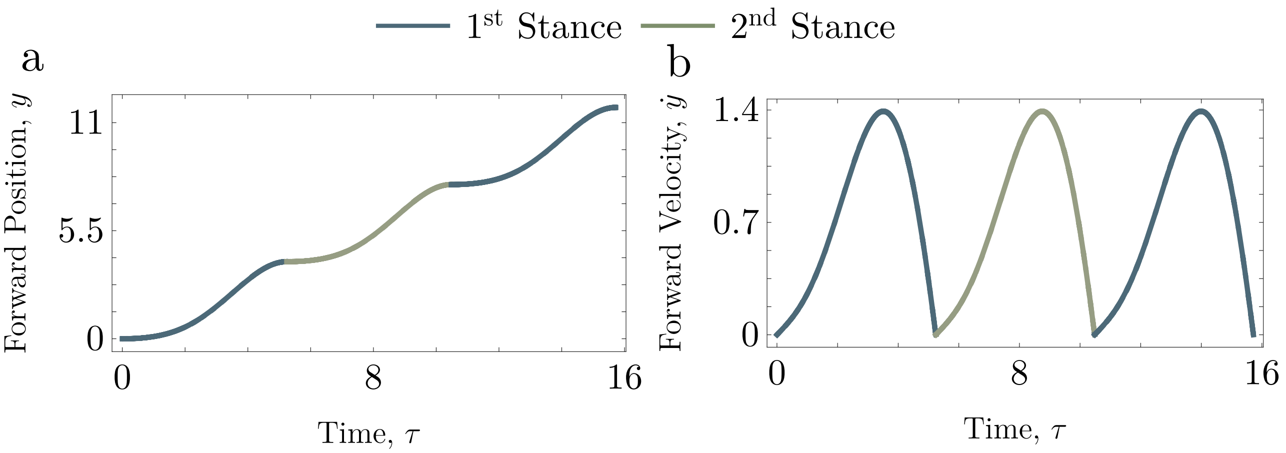

Figure S10: Example walking trajectory for the simplified model. Non-dimensional forward position, y, (a) and velocity, $\dot{y}$, (b), of the CoM for three steps in the simplified model as a function of non-dimensional time, τ (a). We use parameter combination $\varphi = 0.7$, $\dot{y}_0 = 0$, and $\ell'_0 = 0.4$. The blue and green segments of the curves identify the alternating first and second stance phase of the model, respectively.

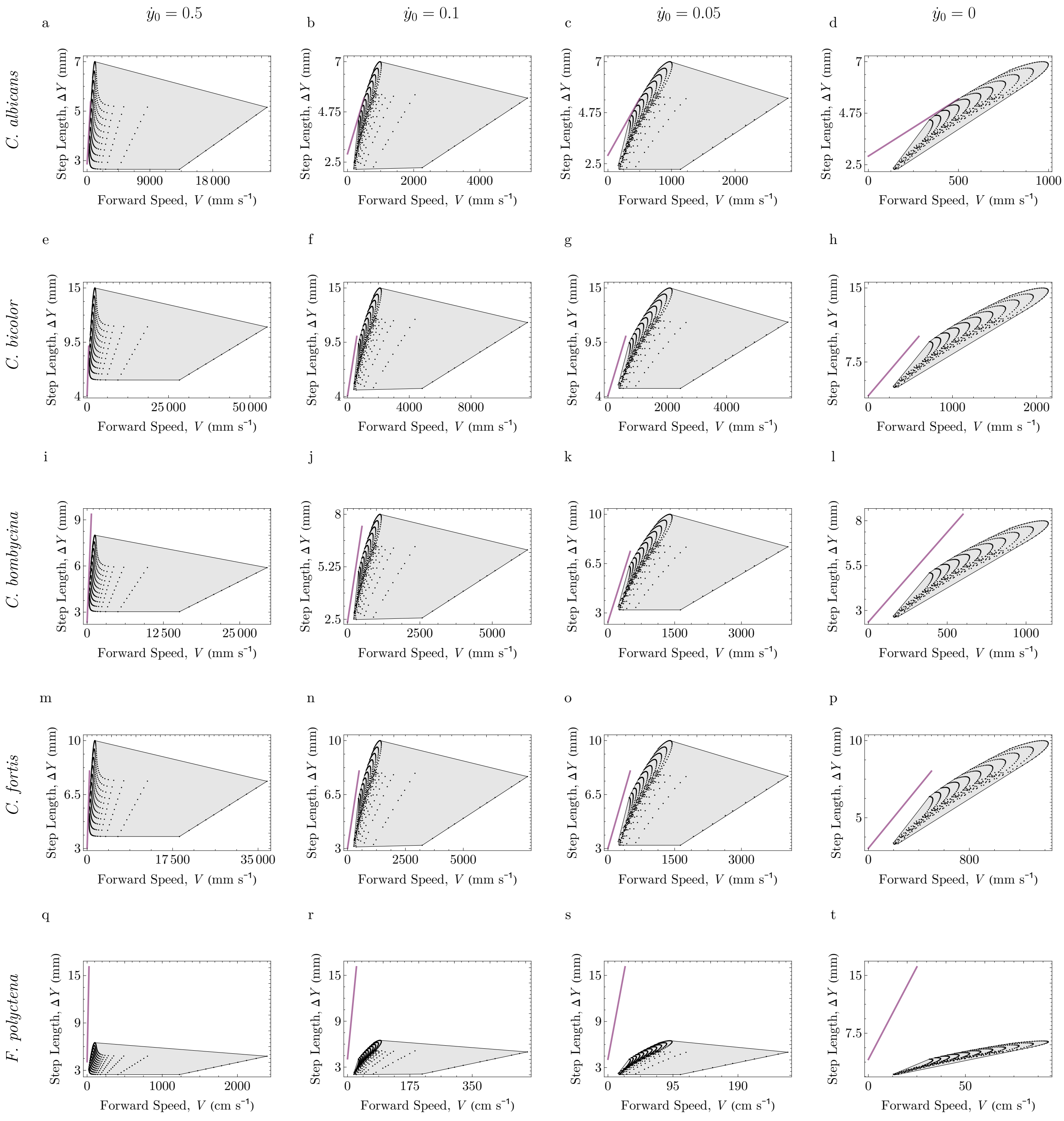

Figure S11: Validation of the simplified model against experimental trends from the literature [4, 5, 10]. The influence of the initial velocity in capturing experimental trends is investigated for *C. albicans* (a-d), *C. bicolor* (e-h), *C. bombycina* (i-l), *C. fortis* (m-p), and *F. polyctena* (q-t). ℓ_0' is varied from 0 to 1 with an increment of 0.1, and φ is varied from 0.01 to 1 with an increment of 0.02. At each point in the (ℓ_0', φ)-domain, we compute the forward speed and step length corresponding to that (ℓ_0', φ)-pair. Then, using maximum leg lengths, $L_{\max}$, of 5.36 mm, 10 mm, 7 mm, 8.25 mm, and 5.39 mm for *C. albicans* (a-d), *C. bicolor* (e-h), *C. bombycina* (i-l), *C. fortis* (m-p) and *F. polyctena* (q-t), respectively, we rescale each point in the non-dimensional region to the forward speed versus step length domain. The forward speed–step length relationship becomes more reliable at high speeds for *Cataglyphis*. For *F. polyctena*, changing the initial velocity is not sufficient for the model to capture experimental trends.

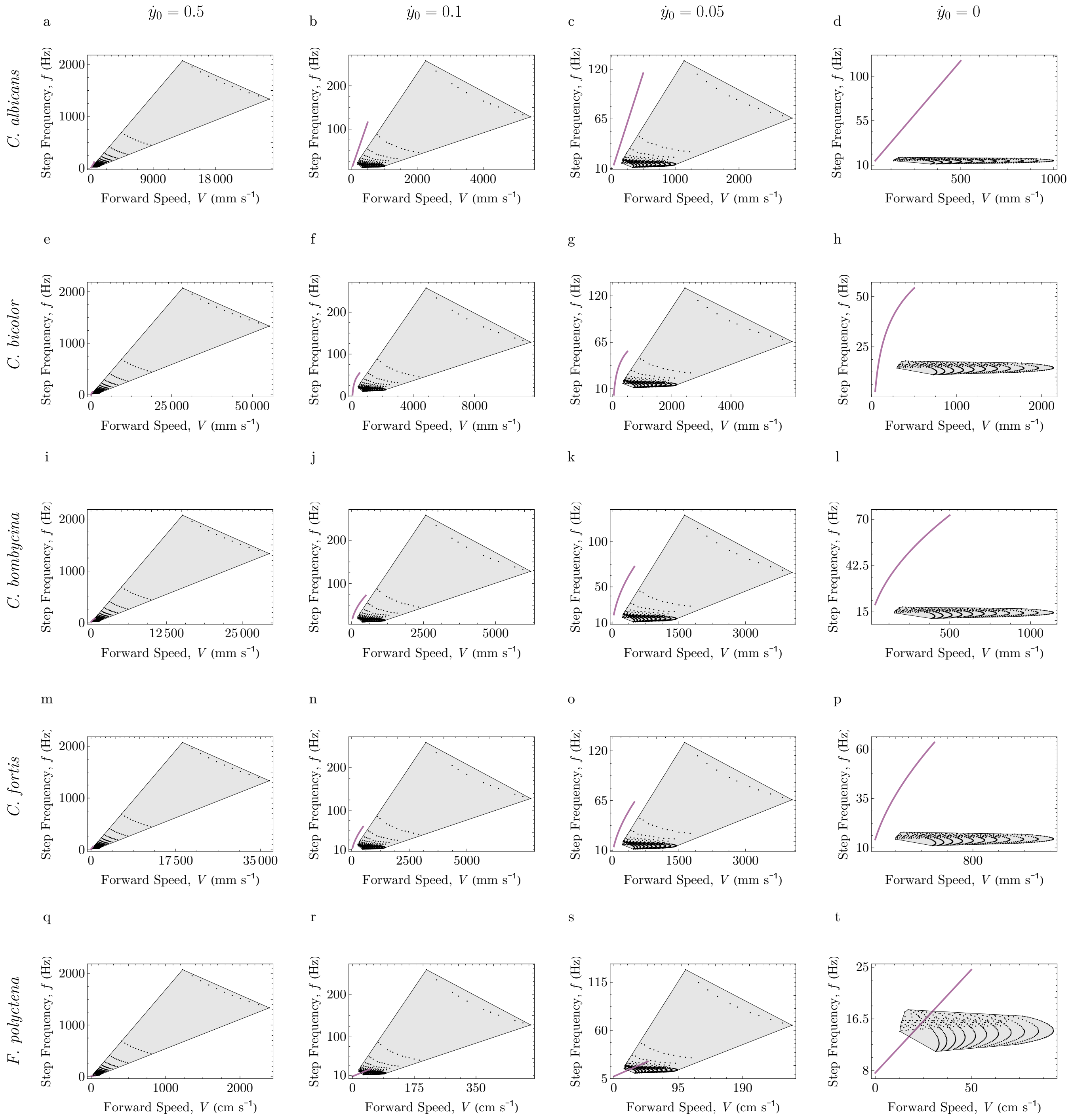

Figure S12: Validation of the simplified model against experimental trends from the literature [4, 5, 10]. The influence of the initial velocity in capturing experimental trends is investigated for *C. albicans* (a-d), *C. bicolor* (e-h), *C. bombycina* (i-l), *C. fortis* (m-p), and *F. polyctena* (q-t). ℓ_0' is varied from 0 to 1 with an increment of 0.1, and φ is varied from 0.01 to 1 with an increment of 0.02. At each point in the (ℓ_0', φ)-domain, we compute the forward speed and step frequency corresponding to that (ℓ_0', φ)-pair. Then, using maximum leg lengths, $L_{\max}$, of 5.36 mm, 10 mm, 7 mm, 8.25 mm, and 5.39 mm for *C. albicans* (a-d), *C. bicolor* (e-h), *C. bombycina* (i-l), *C. fortis* (m-p) and *F. polyctena* (q-t), respectively, we rescale each point in the non-dimensional region to the forward speed versus step frequency domain. For any initial velocity range, the simplified model poorly captures the step frequency of *Cataglyphis*. However, there is an overlap between the forward speed-step frequency trend and the model in *F. polyctena* at an initial forward velocity of zero.

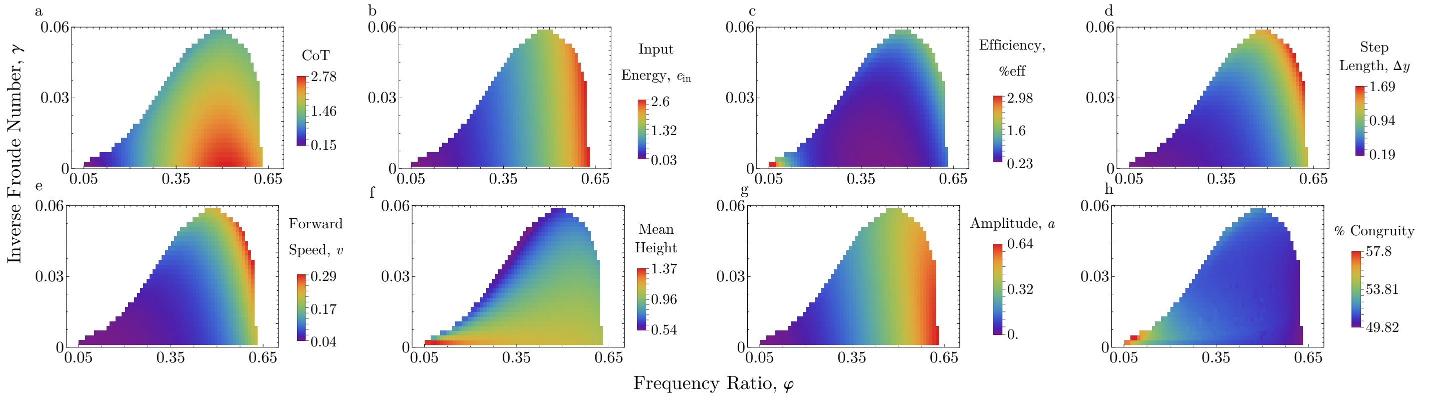

Figure S13: Effect of damping on non-dimensional energetics and gait characteristics in the model as functions of the parameters φ and γ. We hold all other parameters constant at values of $z_0 = 1$, $\dot{z}_0 = 0$, $\dot{y}_0 = 0.04$, $\ell_\text{d} = 1.5$, $\delta y = 0.05$, and $\zeta = 0.05$. We observe minimal variation from the undamped model for each of the measured trends: CoT (a), input energy (b), energy efficiency (c), step length (d), forward speed (e), mean height (f), amplitude (a), and percentage congruity (h). These results suggests that the undamped model presented in the main text is sufficient for predicting the order-of-magnitude of the energy consumption of individual ants. However, a more robust experimental analysis is required to draw more general claims on the necessity of a damping parameter in the model.

References

[1] Thomas Endlein and Walter Federle. On heels and toes: How ants climb with adhesive pads and tarsal friction hair arrays. *PLoS ONE*, 10(11):e0141269, November 2015.

[2] Sumihiro Kohyama, Hidetoshi Takahashi, Tomoyuki Takahata, and Isao Shimoyama. High sensitive and large area force plate for ground reaction force measurement of ant running. In *2018 IEEE Micro Electro Mechanical Systems (MEMS)*, pages 874–877, January 2018.

[3] Hugo Merienne, Gérard Latil, Pierre Moretto, and Vincent Fourcassié. Dynamics of locomotion in the seed harvesting ant *Messor barbarus*: Effect of individual body mass and transported load mass. *PeerJ*, 9:e10664, January 2021.

[4] Sarah Elisabeth Pfeffer, Verena Luisa Wahl, Matthias Wittlinger, and Harald Wolf. High-speed locomotion in the Saharan silver ant, *Cataglyphis bombycina*. *Journal of Experimental Biology*, 222(20), October 2019.

[5] Lars Reinhardt and Reinhard Blickhan. Level locomotion in wood ants: evidence for grounded running. *Journal of Experimental Biology*, 217(13):2358–2370, January 2014.

[6] Lars Reinhardt, Tom Weihmann, and Reinhard Blickhan. Dynamics and kinematics of ant locomotion: Do wood ants climb on level surfaces? *Journal of Experimental Biology*, 212(15):2426–2435, August 2009.

[7] Steven H. Strogatz. *Nonlinear Dynamics and Chaos*. CRC Press, 0 edition, May 2018.

[8] Hidetoshi Takahashi, Sumihiro Kohyama, Tomoyuki Takahata, and Isao Shimoyama. MEMS highly sensitive and large-area force plate for total ground reaction force measurement of running ant. *Journal of Micromechanics and Microengineering*, 34(4):045006, April 2024.

[9] Hidetoshi Takahashi, Nguyen Thanh-Vinh, Uijin G Jung, Kiyoshi Matsumoto, and Isao Shimoyama. MEMS two-axis force plate array used to measure the ground reaction forces during the running motion of an ant. *Journal of Micromechanics and Microengineering*, 24(6):065014, June 2014.

[10] Johanna Tross, Harald Wolf, and Sarah Elisabeth Pfeffer. Allometry in desert ant locomotion (*Cataglyphis albicans* and *Cataglyphis bicolor*) - Does body size matter? *Journal of Experimental Biology*, 224(18), September 2021.

[11] Toni Wöhrl, Lars Reinhardt, and Reinhard Blickhan. Propulsion in hexapod locomotion: How do desert ants traverse slopes? *Journal of Experimental Biology*, 220(9):1618–1625, January 2017.
